# LINC MIR503HG Controls SC‐β Cell Differentiation and Insulin Production by Targeting CDH1 and HES1

**DOI:** 10.1002/advs.202305631

**Published:** 2024-01-20

**Authors:** Yang Xu, Susu Mao, Haowen Fan, Jian Wan, Lin Wang, Mingyu Zhang, Shajun Zhu, Jin Yuan, Yuhua Lu, Zhiwei Wang, Bin Yu, Zhaoyan Jiang, Yan Huang

**Affiliations:** ^1^ Department of Hepatobiliary and Pancreatic Surgery Affiliated Hospital of Nantong University Medical School of Nantong University Nantong 226001 China; ^2^ Center of Gallbladder Disease Shanghai East Hospital Institute of Gallstone Disease School of Medicine Tongji University Shanghai 200092 China; ^3^ Research Center of Clinical Medicine Affiliated Hospital of Nantong University Medical School of Nantong University Nantong 226001 China; ^4^ Key Laboratory of Neuroregeneration of Jiangsu and Ministry of Education NMPA Key Laboratory for Research and Evaluation of Tissue Engineering Technology Products Co‐innovation Center of Neuroregeneration Nantong University Nantong 226001 China; ^5^ Department of Graduate School Dalian Medical University Dalian Liaoning 116000 China; ^6^ Department of Nuclear Medicine Beijing Friendship Hospital Affiliated to Capital Medical University Beijing 100050 China; ^7^ Department of Endocrinology and Metabolism Affiliated Hospital of Nantong University Medical School of Nantong University Nantong 226001 China

**Keywords:** differentiation, human pluripotent stem cells, lncRNA, pancreatic progenitors, scRNA‐seq

## Abstract

Stem cell‐derived pancreatic progenitors (SC‐PPs), as an unlimited source of SC‐derived β (SC‐β) cells, offers a robust tool for diabetes treatment in stem cell‐based transplantation, disease modeling, and drug screening. Whereas, PDX1^+^/NKX6.1^+^ PPs enhances the subsequent endocrine lineage specification and gives rise to glucose‐responsive SC‐β cells in vivo and in vitro. To identify the regulators that promote induction efficiency and cellular function maturation, single‐cell RNA‐sequencing is performed to decipher the transcriptional landscape during PPs differentiation. The comprehensive evaluation of functionality demonstrated that manipulating LINC MIR503HG using CRISPR in PP cell fate decision can improve insulin synthesis and secretion in mature SC‐β cells, without effects on liver lineage specification. Importantly, transplantation of MIR503HG^−/−^ SC‐β cells in recipients significantly restored blood glucose homeostasis, accompanied by serum C‐peptide release and an increase in body weight. Mechanistically, by releasing CtBP1 occupying the CDH1 and HES1 promoters, the decrease in MIR503HG expression levels provided an excellent extracellular niche and appropriate Notch signaling activation for PPs following differentiation. Furthermore, this exhibited higher crucial transcription factors and mature epithelial markers in CDH1^High^ expressed clusters. Altogether, these findings highlighted MIR503HG as an essential and exclusive PP cell fate specification regulator with promising therapeutic potential for patients with diabetes.

## Graphical abstract

The prospect of clinical applications and basic medical research of hESCs derived β cells is limited by imperfect differentiation efficiency, off‐target and unexpected cell byproducts, and insufficient insulin production. Manipulated LINC MIR503HG by CRISPR in hESCs improves insulin synthesis and secretion in later differentiated mature SC‐β cells, without effects on liver lineage specification.

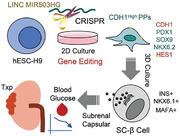

## 1 Introduction

As a global health epidemic, diabetes afflicts an estimated 537 million people worldwide due to β cell deficiency or dysfunction.^[^ ^1^ ^]^ The generation of human pluripotent stem cell (hPSC)‐derived functional pancreatic β cells may solve the problem of cadaveric donor islet supply shortage for transplantation and it could also function as a model platform to shed light on the pathogenic mechanisms leading to various forms of diabetes^[^ ^2^ ^]^ Encouraging progress has been achieved in prolonged in vitro hPSC differentiation toward mature and functional pancreatic β cells by mimicking the in vivo normal developmental trajectory following less than 20 years of efforts.^[^ ^3^ ^]^ However, imperfect differentiation efficiency, off‐target and unexpected cell byproducts, multi‐hormone cell production, and current cost‐ and time‐consuming approaches limit the prospects of clinical applications and basic medical research.^[^ ^3^, ^4^ ^]^ Currently, advanced in vitro differentiation protocols are based on at least six stages, initiating cells from the definitive endoderm (DE), which then undergo the primitive gut tube (PG), posterior foregut (PFG), pancreatic progenitor (PP), and endocrine progenitor (EP) stages before resulting in β cells.^[^ ^3^, ^5^ ^]^ As a key intermediate cell type during stepwise development, PPs give rise to the primary pancreatic, acinar, ductal, and endocrine cell lineages.^[^ ^5a^ ^]^ Moreover, several genetic studies reported that mutations in PPs result in neonatal diabetes and block β cell differentiation from human pluripotent stem cells.^[^ ^6^ ^]^ Therefore, a potential solution for such problems, aiming to obtain functional and clinically valuable human pancreatic cells for diabetes treatment, would be the upscaled generation of high‐quality PPs, developmentally closer to β cells.^[^ ^6b^ ^]^

Long non‐coding RNAs (lncRNAs) comprise a significant part of the human transcriptome and rarely encode proteins.^[^ ^7^ ^]^ Furthermore, they reportedly contribute to differentiation and cell fate specification. For example, the lncRNA Hoxa1 cooperates with Halr1 to regulate the RA signaling pathway and orchestrates embryonic stem cell (ESC) endoderm differentiation.^[^ ^8^ ^]^ Meteor, a mesendoderm progenitor lncRNA expressed in eomesodermin (*EOMES*), interacts with *EOMES* and epigenetically functions as a regulator of mesendoderm specification and cardiac differentiation.^[^ ^9^ ^]^ Inhibition of MAPT213 synthesis promotes processive MAPT transcription, a key neuronal gene involved in neural stem cell differentiation into neurons, through ZMYND8 association with transcription repressor complexes.^[^ ^10^ ^]^ However, roles and molecular mechanisms of lncRNAs during the stem cell‐derived β (SC‐β) cell specification and differentiation of the PP lineage remain elusive.

To identify pancreatic‐specific lncRNAs that control PP cell fate and reveal the underlying regulatory mechanisms, we carried out single‐cell RNA‐sequencing (scRNA‐seq) analysis in PG, PFG, and PP cells as a continuous developmental process. Subpopulation‐specific MIR503HG drew our attention, which reportedly functions in endothelial‐to‐mesenchymal transition (EndMT) in pulmonary hypertension, suppress hepatocellular carcinoma metastasis by promoting HNRNPA2B1 ubiquitination.^[^ ^11^ ^]^, and plays a role in PP differentiation and maturation by targeting the CDH1 and HES1 promoters. E‐cadherin (E‐cad) reportedly initiates cell signaling through intracellular pathway activation involving protein phosphorylation and can modulate cell differentiation, polarity, proliferation, and cell‐cell communications beyond contributing to epithelial structure and integrity.^[^ ^12^ ^]^ CDH1, a PDX1 target in the rodent pancreas, has recently been confirmed to promote insulin (INS) secretion, resist apoptosis, glucotoxicity, lipotoxicity, and endoplasmic reticulum stress through a complex interaction with signaling pathways.^[^ ^13^ ^]^ HES1, downstream of the Notch pathway, regulates the determination between the progenitor state and cell fate commitment to the transition toward the differentiation and maturation states in nearly every tissue.^[^ ^14^ ^]^ During pancreatic development, PPs maintain their proliferation and multipotency by upregulating HES1 transcription and translation.^[^ ^15^ ^]^ As development proceeds, HES1 repression allows cells to differentiate toward the pancreatic endocrine cell fate, whereas PPs maintain the progenitor state and direct cells toward a pancreatic ductal fate by maintaining SOX9 expression.^[^ ^16^ ^]^

In the current study, we investigated the PP‐specific lncRNA MIR503HG, which functions by recruiting CtBP1 to modulate downstream target genes CDH1 and HES1, in following differentiation into SC‐β cells. We introduced two lncRNA MIR503HG mutations (MIR503HG^−/−^) into the genome of H9 human ESCs (hESCs) differentiated into SC‐β cells. The generated MIR503HG^−/−^ SC‐β cells displayed enhanced INS‐producing capacity and significantly improved glycemic control in diabetic mice, offering an advanced strategy for stem cell‐based cell replacement therapy for diabetes.

## 2 Experimental Section

### 2.1 Cell Culture and Animals

The hESC‐H9 line used in SC‐β cells differentiation protocols and other experiments was obtained from Professor Yan Liu of Nanjing Medical University. hESCs were cultured with complete mTeSR™ Plus medium (100‐0276, STEMCELL Technologies) 5% CO_2_ at 37°C. The planar culture of hESCs were described previously.^[^ ^17^ ^]^ Male SCID‐Beige mice were purchased from GemPharmatech Company and maintained in the animal facility of Nantong University. All animal studies were approved by the Animal Ethics Committee of Nantong University (S20220219‐004). This study conformed with the Helsinki Declaration of 1975, as revised in 2008 (5) concerning Human and Animal Rights.

### 2.2 Human Pancreatic and Hepatic Differentiation from hPSC‐H9 Line

For pancreatic differentiation, two days after passaging, cells seeded onto 6‐well plates coated with Matrigel (354277, Corning) at 3 × 10^5^ cells per cm^2^ were differentiated with an adapted 6‐stage protocol according to previous studies.^[^ ^3^, ^18^ ^]^ Stage 1 (5 days): DE, Stage 2 (3 days): PG, Stage 3 (3 days): PFG, Stage 4 (5 days): PP, Stage 5 (7 days): EP, Stage 6 (4 days): stem cells derived‐β cells (SC‐β cells). Details for differentiation protocol was described in Table S1 (Supporting Information).

For hepatic differentiation, two days after passaging, cells seeded onto 6‐well plates coated with Matrigel (354277, Corning) at 2 × 10^5^ cells per cm^2^ were differentiated with a 3‐stage protocol based on STEM diff™ Hepatocyte Kit (100‐0520, StemCell technologies) protocol. Stage 1 (5 days): DE, Stage 2 (5 days): hepatic progenitor (HP), Stage 3 (11 days): stem cells derived‐hepatocyte (SC‐hepatocytes).

### 2.3 Generation of MIR503HG Knockout hES‐H9 Line

The sgRNAs targeting the human MIR503HG was designed (www.genome engineering.org/crispr) and inserted into the vector carrying a Cas9 gene (YKO‐RP003‐MIR503HG.[gRNA3‐gRNA4]) and a resistance cassette for puromycin. The construct (1µg) was electroporated into 5 × 10^5^ hES‐H9 cells. Then, the cells were cultured in one well of 6‐well plate with hESC medium containing puromycin (1µg ml^−1^). After 48–72h, 1000–2000 cells were passaged and cultured in 10 cm dishes until the colonies were visible. Colonies were picked, expanded, genotyped, and analyzed by Sanger sequencing or cryopreserved for further studies. Primer sequences and sequences for sgRNAs were listed in Table S2 (Supporting Information).

### 2.4 Alkaline Phosphatase (ALP) Staining

Cells cultured on plates were washed once with PBS and then fixed with 4% paraformaldehyde for 15 min at room temperature (RT). ALP staining was performed using Pluripotent Stem Cell Alkaline Phosphatase Color Development Kit (C3250S, Beyotime) following the manufacturer's instructions. Briefly, cell samples were incubated with prepared BCIP/NBT staining solution for 25 min, followed by washing with ddH_2_O twice. Then, images were pictured with Nikon Ti2‐E microscopes in the bright field.

#### 2.4.1 qRT‐PCR

Total RNA was isolated using the RNA‐easy Isolation Reagent (R701‐01, Vazyme) following the manufacturer's instructions. First‐strand cDNAs for lncRNA, mRNAs and miRNAs were generated using the HiScript II 1st Strand cDNA Synthesis Kit (+gDNA wiper) (R212‐02, Vazyme) and miRNA 1st Strand cDNA Synthesis Kit (by stem‐loop) (MR101‐02, Vazyme) respectively and then the products were used as templates in qRT‐PCR based on SYBR Green (ChamQ Universal SYBR qRT‐PCR Master Mix, Q711‐02, Vazyme). Results were analyzed using a 2^−ΔΔCt^ methodology. Each biological reaction was performed triply. Expression of mRNAs and lncRNA were normalized to GAPDH, miRNAs were to U6. Primers were listed in Table S3 (Supporting Information).

#### 2.4.2 Immunofluorescence (IF)

Cells cultured on plates were washed once with PBS and then fixed with 4% paraformaldehyde for 15 min at room temperature (RT). After being washed with PBS, the samples were permeabilized with PBS with 0.3% TritonX‐100 for 15 min and then blocked with 5% BSA Blocking Buffer (SW3015, Solarbio) for 120 min. The samples were incubated with the primary antibody diluted with permeabilization solution overnight at 4°C, then being washed with PBS for three times, further incubated with secondary antibodies for 1h at RT, and stained with DAPI. Images were taken using Leica SP8 and Nikon Ti2‐E microscopes. Primary and secondary bodies and solution ratio were listed in Table S4 (Supporting Information).

#### 2.4.3 Western Blotting (WB)

Cells protein was isolated based on RIPA lysis buffer (PC101, Epizyme) supplemented with 1% Protease Inhibitor Cocktail (GFR101, Epizyme). Protein extracts were separated, resolved on SDS‐PAGE gels, and then transferred to PVDF membranes. PVDF membranes were blocked in BSA for 1h at RT, incubated with primary antibodies overnight at 4°C, and then corresponding secondary antibodies after washing. Membranes were detected by immunoblotting with the Omni‐ECL™Femto Light Chemiluminescence Kit (SQ201, Epizyme) using ChemiDoc MP Imaging system (BIO‐RAD). Antibodies for Western blotting are outlined in Table S4 (Supporting Information).

#### 2.4.4 RNA Immunoprecipitation (RIP)

PPs were harvested and then resuspended in 1 mL lysis buffer (25 mm Tris‐HCl (ST774, Beyotime), pH 7.4, 150 mm NaCl, 0.05 mm DTT, 5 mm EDTA (C0196, Beyotime), 0.5% NP‐40 (P0013F, Beyotime)) containing 100U mL^−1^ RNase Inhibitor (R0102, Beyotime) followed by 3 × 10 s sonication with an interval of 30 s. After centrifuging at 12,000 rpm for 15 min at 4°C. The supernatant was incubated with 5 µg anti‐CtBP1 antibody (8684S, Cell Signaling Technology) or anti‐IgG antibody (3900S, Cell Signaling Technology) for 4h at 4°C. 50 µl pre‐balanced Protein A/G Magnetic Beads (HY‐K0202, MCE) were added to the mixture and incubated for 1h at 4°C. Then, the mixture was washed with wash buffer followed by eluting with RNA‐easy Isolation Reagent (R701‐01, Vazyme) at room temperature for 10 min. RNA was isolated as described above. qRT‐PCR was performed for RNA expression analysis. The anti‐IgG antibody was as negative control and total RNAs was for input. Primers were listed in Table S3 (Supporting Information) and antibodies for Western blotting are outlined in Table S4 (Supporting Information).

#### 2.4.5 mRNA‐seq

Total RNA was isolated from all samples using the RNA‐easy Isolation Reagent (R701‐01, Vazyme) following the manufacturer's instructions. cDNA library construction and high throughput sequencing were carried out by Novogene. The differential expression analysis, Gene Ontology (GO), and Kyoto Encyclopedia of Genes and Genomes (KEGG) enrichment analyses were performed using Omicsmart (www.omicshare.com/tools/).

#### 2.4.6 scRNA‐Seq and Data Analysis

For sequencing, library synthesis and scRNA‐seq were carried out by Gene Denovo (Gene Denovo, China) as described previously.^[^ ^17^ ^]^ Briefly, cells were dissociated into single cells with Accutase (07920, STEMCELL Technologies), then resuspended in PBS. Next, cell mixture was filtrated via a 40‐µm cell strainer (352340, Corning) and diluted to a concentration of 10,000 cells/ml. Single cells were isolated in emulsions followed by each cell was label‐barcoded with a unique set of oligonucleotides. PCR was performed to amplify the complementary DNA (cDNA) library. All groups of cDNA libraries were pooled on the Illumina 10 × Genomics Chromium platform (10 × GENOMICS, Illumina) and sequenced with paired‐end reads.

For data analysis in brief, the cell‐by‐gene matrices were produced by UMI counting and cell barcodes calling and then individually imported to Seurat version 3.1.1 for identification of cell clusters. Cells with over 8000 UMIs, less than 500 or more than 4000 genes, and over 10% mitochondrial gene percent were filtered out. Principal component analysis (PCA) was performed for dimensional reduction. An approach for graph‐based clustering was implemented by Seurat. Briefly, Seurat embed cells in a shared‐nearest neighbor (SNN) graph, with edges drawn between cells via similar gene expression patterns. After constructing the SNN graph based on the euclidean distance in PCA space, the edge weights between any two cells were refined according to the common overlap and refined the edge weights between any two cells based on Jaccard distance. Generation of clusters was based on Louvain method, which was visualized by t‐distributed Stochastic Neighbor Embedding (t‐SNE) using the same PCs.

### 2.5 Pull Down

The full‐length MIR503HG sequence was PCR amplified using a T7‐containing primer and then reversely transcribed by MAXIscriptTM T7 Transcription Kit (AM1312, Thermo Fisher Scientific). The targeted RNA was Biotin‐labeled with PierceTM RNA 3’ End Desthiobiotinylation Kit (20163, Thermo Fisher Scientific). PPs were harvested and added with protein lysis buffer and then the biotin‐labeled MIR503HG probe was captured using Streptavidin magnetic beads (P2151, Beyotime). The biotinylated nucleic acid compounds were incubated with the protein extract from PPs by Pierce^TM^ Magnetic RNA‐Protein Pull‐Down Kit (20164, Thermo Fisher Scientific). Beads were washed five times and then boiled in SDS buffer. Next, supernatant was further for Mass spectrometry and Western blotting. The extracted protein was designated as the positive control, whereas the antisense RNA was used as the negative control. Antibodies for Western blotting are outlined in Table S4 (Supporting Information).

### 2.6 Mass Spectrometry

Samples pulled down were measured on SDS‐PAGE gels which was stained with Fast Silver Stain Kit (P0017S, Beyotime) following the manufacturer's instructions. LCMS/MS was carried out to analyze specific bands. Then retrieval of protein identification was performed in the human RefSeq protein database (National Center for Biotechnology Information, NCBI).

### 2.7 C‐Peptide Content Analysis

The C‐peptide content of mice transplanted with SC‐β cells was analyzed as we reported previously. Venous blood of mice was collected and centrifuged, then the Human C‐peptide content was assessed using an ultrasensitive human C‐peptides assay kit (10‐1141‐01, Mercodia) following the manufacturer's instructions.

#### 2.7.1 Glucose‐Stimulated Insulin Secretion (GSIS)

After pre‐incubation, in Krebs–Ringer Bicarbonate buffer (KRB) for 2 h to remove residual insulin, the SC‐β cells were treated by KRB containing low (2.8 mm) or high (16.7 mm) glucose respectively for 30 min. Then, supernatants were collected and then the pellets were dispersed into single cells using Accutase (07920, STEMCELL Technologies) for cell counting using a cell counter (C100‐SE, RWD). The same procedures were performed for stimulating cells with 30 mm KCl, 200 µm tolbutamide, or 10 nm exendin‐4. Insulin was measured by a human insulin ELISA kit (10‐1113‐01, Mercodia) following the manufacturer's instructions.

#### 2.7.2 Intraperitoneal Glucose Tolerance Tests (IPGTT)

After a 12 h fast, 3 g/kg of 30% glucose was infused i.p. into the mice within 1 min. Blood glucose concentrations were measured with a handheld glucometer at 0, 30, 60, 90, and 120 min after injection.

### 2.8 Human Albumin and Urea Content Analysis

Human albumin was measured using the Human Albumin ELISA kit (SEKH‐0081, Solarbio) according to the manufacturer's instructions. Urea synthesis was examined with the Urea Assay Kit (KA1652, Abnova) according to the manufacturer's instructions.

#### 2.8.1 Flow CytoMetry (FCM)

Cells were dissociated into single cells using Gentle Cell Dissociation Reagent (100‐0485, STEMCELL Technologies). Then, cells were fixated and permeabilized at the same time via Fixation/Permeabilization solution (554714, BD Bioscience) for 30 min and followed by centrifuged at 300 × g for 5 min. Thereafter, cells were incubated with Perm/Wash buffer (554714, BD Bioscience). After washing with Perm/Wash buffer once, cells were incubated in antibodies at 4°C for 40 min. Samples were analyzed using LSRFortessa^TM^ X‐20 (BD Biosciences), and data was analyzed using Flowjo (7.0, Flowjo Software). All antibodies are detailed in Table S4 (Supporting Information).

### 2.9 EdU

Cell proliferation was investigated with BeyoClickTM EdU Cell Proliferation Kit (C0075S, Beyotime) as per the manufacturer's instructions. In brief, proliferating PPs were incubated with Edu working solution (10 µm) for 2 h at 37°C avoiding the light. After incubation, PPs were fixed with 4% paraformaldehyde and then permeabilized with 0.5% TritonX‐100 both for 15 min. Then, cells were co‐stained with C‐peptide or PDX1 following steps in Immunostaining. The images were captured with Nikon Ti2‐E microscope.

### 2.10 Cytoplasmic and Nuclear RNA Fractionation

Cytoplasmic and nuclear RNA were extracted from PPs using PARIS^TM^ kit (AM1921, Thermo Fisher Scientific) according to the manufacturer's instructions. RNA extracted from the two fractions was evaluated by qRT‐PCR. Data were analyzed to evaluate the percentages of nuclear and cytoplasmic RNA.

#### 2.10.1 Fluorescence In Situ Hybridization (FISH)

After fixation and permeabilization as the same in Immunostaining, PPs were pre‐hybridized at 37°C for 30 min. Then, hybridization was performed at 42°C overnight, and cellular DNA was finally stained with DAPI for 10 min at RT. Fluorescence signals were detected with Leica SP8. lncRNA MIR503HG‐cy3 FISH probes were designed and synthesized by RiboBio Co., Ltd. U6 and 18S FISH probes were used as the nuclear and cytoplasmic controls respectively.

### 2.11 Karyotype Analysis

The MIR503HG knockout hES‐H9 cells were cultured until reaching 80% confluency. Colchicine (ST1173, Beyotime) was added to the medium and incubated for 3 h at 37°C, and then cells were digested into single cell by Accutase (07920, STEMCELL Technologies). The cells were resuspended in 75 mm KCl solution for 30 min and then incubated in a fixative containing ethanol/acetic acid (3:1, v/v) for 20 min at RT. 20 µl cells suspension were applied to each clean slide. Dyed by Giemsa stain (C0131, Beyotime) for 30 min at RT, 20 metaphases were counted for each sample, and then chromosome analysis were performed using the Leica DMRB epifluorescence microscope.

### 2.12 Electron Microscopy

SC‐β cell clusters were harvested, washed with PBS, and fixated with pre‐cold fixative (G1102, Servicebio) for 2 h. Then, clusters were embedded with agarose (1% v/v). The samples were further processed following a protocol for electron microscopy sample by the Electron Microscopy Facility of the Jiangsu Key Laboratory of Neuroregeneration, Nantong University. The images of insulin granules were captured using a transmission electron microscope (Hitachi, HT7700).

### 2.13 Transplantation of SC‐β Cell Clusters

Transplantation of SC‐β cell clusters and STZ‐induced diabetic SCID‐Beige mice were performed as described previously.^[^ ^17^ ^]^ ≈ 2 × 10^6^ MIR503HG^−/−^ or WT SC‐β cell clusters were transplanted into the capsule kidney of every male SCID‐Beige mice or STZ‐induced SCID‐Beige nude diabetic mice aged 12 weeks at the animal center of Nantong University. Kidneys with grafts were harvested and fixed in 4% PFA after 15 weeks. Then the fixed samples were embedded and sectioned followed by Hematoxylin or eosin (H&E) and IF analysis.

### 2.14 ChIP‐qRT‐PCR

ChIP assay for PPs was performed with an EZ‐Magna CHIP assay kit (17‐10086, Merck) according to the manufacturer's instructions. Briefly, PPs were harvested, cross‐linked with 1% formaldehyde, lysed, and then sonicated. The DNA‐protein complexes were then isolated with appropriate antibodies which were listed in Table S2 (Supporting Information). The primers were designed according to the promoter sequences of CDH1 or HES1. The sequences of primers are shown in Table S3 (Supporting Information).

### 2.15 Statistical Analysis

Data were derived from at least three independent replicates. P values were calculated by two‐tailed unpaired Student's *t*‐test or one‐way ANOVA analysis using GraphPad Prism 9 software. *p* < 0.05 was considered significant statistically.

## 3 Results

### 3.1 lncRNA Landscape during Human PP Differentiation

**Figure 1:**
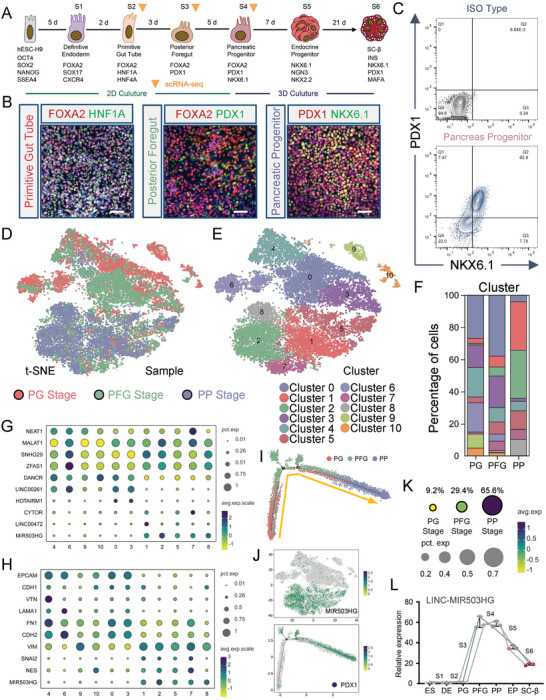
lncRNA landscape during human PP cell differentiation. A) Schematic representation of differentiation protocol imitating human pancreatic β cell development. B) IF of PG, PFG, and PP cells for corresponding markers. Scale bar: 100 µm. C) FCM performed on PP cell populations for PDX1 and NKX6.1. D) *t*‐SNE projection from unsupervised clustering of scRNA‐seq of PG, PFG, and PP cell transcriptional data. E) *t*‐SNE visualization of cells, colored by cell type. F) Calculated percentage of defined PG, PFG, and PP cell clusters. G,H) Dot plot showing DEGs in different cell clusters. Color and size of each dot represent the expression level and percentage of cells expressing the given gene. I) Distribution of pseudotime of PG, PFG, and PP cells. J) Upper panel: *t*‐SNE visualization of cells based on the expression of MIR503HG expression. Lower panel: Distribution of pseudotime of PG, PFG, and PP cells based on expression of PDX1 expression. K) Calculated percentage and expression levels of MIR503HG expressed PG, PFG, and PP cells. L) qRT‐PCR analysis of MIR503HG expression in cells at different stages. qRT‐PCR, FCM, and staining results were repeated in at least three independent differentiations. All data are expressed as means ± SD. Statistical significance was calculated using a two‐tailed Student's *t*‐test.

We have previously reported on the transcriptional profiling of bulk populations from mouse‐induced PSC (miPSC)‐derived insulin‐producing cells, focusing on microRNAs and lncRNAs.^[^ ^19^ ^]^ We adopted an improved stepwise differentiation protocol to generate human pancreatic β cells from hESCs (**Figure** **1**A) and applied immunofluorescence (IF) and flow cytometry (FCM) to assess the differentiation process (Figure 1B,C). After pancreatic specification, the FCM results indicated that over 60% of the cells were PDX1‐ and NKX6.1‐double positive, suggesting successful differentiation (Figure 1C). In this study, we were intrigued about whether subset‐specific lncRNA could precisely regulate cell fate specification. To investigate single‐cell lncRNA dynamics during PP differentiation, we performed scRNA‐seq at the S2 (PG), S3 (PFG), and S4 (PP) stages (Figure 1A). The cells in the 3 stages (19746 PGs, 20520 PFGs, and 17538 PPs) were classified into 11 clusters using t‐distributed stochastic neighbor embedding (t‐SNE), among which the proportions of Cluster 1 and 2 rose significantly as the differentiation progressed (Figure 1D–F). Some crucial transcription factors (TFs) for pancreatic lineage specification are distributed among most of the SC‐PPs clusters, including PDX1, NKX6.1, SOX9, ONECUT1/2, GATA6, and FOXA2 (Figure S1A, Supporting Information). Next, we performed pseudotime analysis to construct the differentiation process from PG to PP. After cell identity annotation and summarizing the distribution of the cells of in the 3 stages along the pseudotime trajectory, Cluster 1, 2, and 7 were confirmed as the end of the differentiation trajectory (Figure S1B, Supporting Information). Furthermore, the cells of Cluster 1, 2, and 7 revealed a higher number of active regulons than those of the other clusters (Figure S1C, Supporting Information), some of which reportedly drive gene networks to participate in transcription and signaling pathway regulation during islet development, such as MAF, JUN (c‐JUN/AP‐1), TCF4, CEBPB, ZMIZ1, and BCL11A (Figure S1D,E, Supporting Information).

Next, we plotted several differentiation‐related lncRNAs and partial differentially expressed genes (DEGs).^[^ ^20^ ^]^ Unlike NEAT1, MALAT1, SNHG29, ZFAS1, and DANCER, which were expressed in all cells, LINC00261 and HOTAIRM1, as well as epithelium marker genes including EpCAM, CDH1, VTN, and LAMA1, were enriched in Clusters 4, 6, 9, 10, 0, and 3. Meanwhile, the remaining clusters displayed relatively high expression levels of genes related to neuron differentiation (Nestin.[NES]), cell adhesion (LAMA1 and FN1), and mesenchymal markers (VIM, SNAI2, and CDH2), along with MIR503HG, CYTOR, and LINC00472 (Figure 1G,H). Of note, NES has been reported to function as a pancreatic stem cell marker.^[^ ^21^ ^]^ Furthermore, NES^+^ ESCs or selected NES^+^ cells from adult islets can be converted to INS‐producing cells (IPCs).^[^ ^22^ ^]^ LincRNA00261 and HOTAIRM1 are reportedly indispensable for FOXA2‐dependent endocrine cell development in cis and play a role in PP cell differentiation through exocytosis and retinoic acid receptor signaling pathway regulation, respectively.^[^ ^20e,g^ ^]^ We then focused on the other 3 cluster‐specific lncRNAs, MIR503HG, CYTOR, and LINC00472, which, exhibited expression levels and trends similar to those of the mesenchymal markers (Figure 1G,H). Cell trajectory analysis indicated the specific features of every differentiation stage, and PFGs were the indispensable transit that bridged PGs and PPs (Figure 1I). The *t*‐SNE analysis revealed that MIR503HG was mainly distributed in PPs, in which PDX1 the key TF initiating pancreatic lineage specification begins to express (Figure 1J). The scRNA‐seq results showed that MIR503HG enriched rapidly from the PFGs to the PPs (Figure 1K). And qRT‐PCR data indicated that MIR503HG levels increased first in PFGs, and then peaked in PPs. (Figure 1L; unpublished data). We hypothesized that MIR503HG may act as a regulator in cell adhesion and EMT in PPs during the stepwise hESC‐derived pancreatic β cell differentiation.

### 3.2 MIR503HG^−/−^ H9 Cell Line Establishment using CRISPR/Cas9‐Based Genome Editing

**Figure 2:**
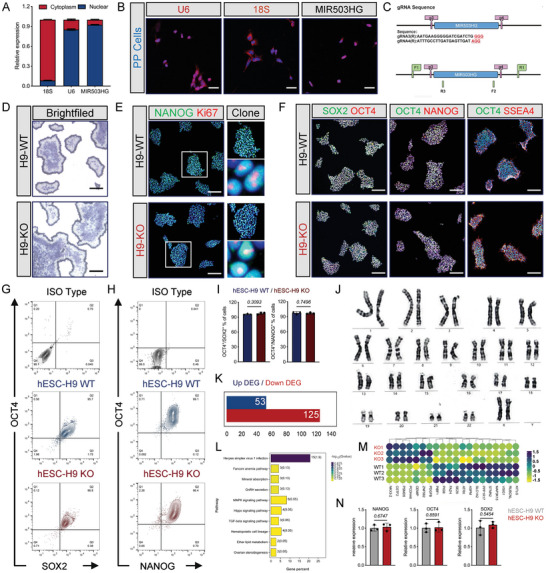
MIR503HG−/− H9 cell line establishment using CRISPR/Cas9‐based genome editing. A) MIR503HG distribution in PP cytoplasm and nucleus. B) FISH of U6, 18S, and MIR503HG in PPs. Scale bars, 50 µm. C) Representative chart of sgRNA for CRISPR/Cas9. D) ALP staining of WT and MIR503HG‐KO ES cells. Scale bar, 500 µm. E) IF for NANOG/KI67 of WT and MIR503HG‐KO ES cells colonies. Scale bars for low magnification, 500 µm; Scale bars for high magnification, 10 µm. F) IF for stem cell markers of WT and MIR503HG‐KO ES cells colonies. Scale bars, 500 µm. G) and H) FCM performed on hESC‐H9 WT and hESC‐H9 KO cell populations for OCT4/SOX2 or OCT4/NANOG, respectively. I) Population percentage of OCT4/SOX2 and OCT4/NANOG double positive cells (n = 3). Data are expressed as means ± SD. J) High‐resolution G‐banding analysis of MIR503HG‐KO ES cells at passage. K) DEGs between WT and MIR503HG‐KO ES cells from mRNA‐seq. L) Exhibition of top 10 KEGG based on DEGs in (K). M) Heatmap for DEGs between WT and MIR503HG‐KO ES cells from mRNA‐seq. N) qRT‐PCR analysis of stem cell markers expression levels. qRT‐PCR, staining, and FCM results were repeated in at least three independent differentiations. All data are expressed as means ± SD. Statistical significance was calculated using a two‐tailed Student's *t*‐test.

To preliminary explore the potential biological role of MIR503HG in pancreatic cell fate specification, qRT‐PCR was performed to examine the expression of pancreas‐associated TFs (Figure S2A, Supporting Information). The expression trend of MIR503HG was opposite to that of some important pancreatic TFs, including GATA6, PAX4, NKX6.1, and UCN3. However, the transcription of some PP TFs such as PDX1 and PTF1A was irrelevant to that of MIR503HG, indicating that MIR503HG may influence the development of pancreatic endocrine cells. Furthermore, MIR503HG knockdown (KD) increased the ratio of PDX1 and NKX6.1‐double positive cells (wild‐type.[WT], 58.95 ± 1.79% vs KO, 73.25 ± 1.93%) and promoted important PP TF and marker expressions, whereas MIR503HG overexpression had nearly no effect on these genes (Figure S2B‐F, Supporting Information). Thus, the regulation of MIR503HG has the potential to promote the differentiation of PPs. whereas subcellular localization was a key characteristic of lncRNA function, both cell fraction analysis and fluorescence in situ hybridization (FISH) indicated that ≈ 90% of transcripts were localized in the nucleus (**Figure** **2**A,B).

Considering its potential for clinical transplantation and the difficulties in ES cell transfection for further studies, we generated MIR503HG knockout (KO) hESC lines using specifically designed sgRNAs and the CRISPR/Cas9 (YKO‐RP003‐MIR503HG.[gRNA3‐gRNA4]) KO strategy (Figure 2C). Two MIR503HG KO hESCs clones (H9‐MIR503HG‐KO1/KO2) were generated (Figure S3A, Supporting Information). Whole sequences of MIR503HG were deleted and effectively validated by Sanger sequencing and PCR (Figures S3B–E and S4, Supporting Information). After gene editing, IF for stemness markers NANOG/OCT4 were similar in KO1 and KO2 ESCs (Figure S3F, Supporting Information). Furthermore, alkaline phosphatase (ALP) staining showed that both KO‐1 and KO‐2 cells had a typical hPSC morphology consistent with those of WT cells during long‐term in vitro culture (Figure S3G, Supporting Information). As differentiation processed into pancreatic progenitors, much more PDX1/NKX6.1 double positive cells were detected in both KO1‐PPs and KO2‐PPs than WT‐PPs via FCM (Figure S3H, Supporting Information, WT vs KO1 vs KO2, 53.7 ± 4.50% vs 72.1 ± 1.26% vs 82.8 ± 2.36%) and IF (Figure S3I, Supporting Information). Otherwise, no MIR503HG transcription was found in KO1‐PPs and KO2‐PPs along with upregulated expression of some TFs for pancreatic lineage specification via qPCR (Figure S3J, Supporting Information). MIR503HG KO2 ESCs was chosen for following biological functions and molecular mechanism studies. We designated the MIR503HG KO and WT hESC lines as H9‐KO and H9‐WT, respectively. ALP staining showed that KO cells had a typical hPSC morphology and proliferation in accordance with those of WT cells during in vitro culture (Figure 2D). IF for pluripotent stem cell markers OCT4, NANOG, SOX2, and SSEA4 and the proliferation marker KI67 showed nearly homogenous expressions of these proteins in both KO and WT cells (Figure 2E,F). FCM analysis for the ratio of OCT4/SOX2 (96.07 ± 1.10% vs 97.70 ± 2.17%) and OCT4/NANOG (97.50 ± 2.33% vs 96.87 ± 1.46%) double positive cells revealed that there was no significance between the two groups (Figure 2G–I). Karyotype analysis revealed no significant mutations in any chromosome after gene editing (Figure 2J). mRNA‐seq analysis indicated that the mRNA expression‐related differences between KO and WT hESCs were insignificant, and the DEGs and enrichment pathways were unrelated to differentiation, proliferation, or stemness (Figure 2K–M). No significant differences were observed in the expression of the stemness markers NANOG, OCT4, and SOX2 by qRT‐PCR (Figure 2N). Taken together, these results suggest that we successfully established an MIR503HG KO line and this gene is dispensable for hESC survival and self‐renewal.

### 3.3 MIR503HG^−/−^ hESCs Efficiently Differentiated into Functional SC‐β Cells In Vitro

**Figure 3:**
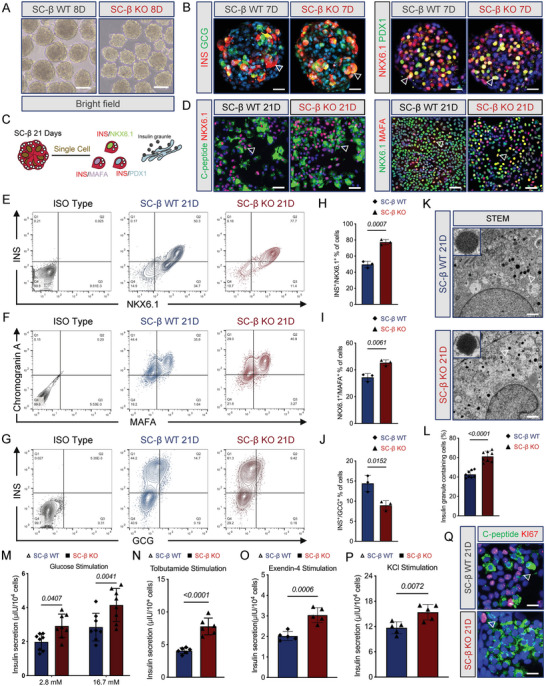
MIR503HG−/− hESCs efficiently differentiated into functional SC‐β cells in vitro. A) Brightfield images of WT and MIR503HG‐KO SC‐β cell clusters. Scale bars, 500 µm. B) IF for INS/GCG and PDX1/NKX6.1 of WT and MIR503HG‐KO SC‐β cell clusters at day 7 of Stage 6. Scale bars, 50 µm. C) Schematic of SC‐β cell clusters digested into single cells and then cultured in culture flasks. D) IF for C‐peptide/NKX6.1 and NKX6.1/MAFA of WT and MIR503HG‐KO SC‐β cell clusters at day 21 of Stage 6. Scale bars, 50 µm. E–G) FCM of WT and MIR503HG‐KO SC‐β cells for INS/NKX6.1, Chromogranin A (CHGA)/MAFA, INS/GCG. H–J) Population percentages of INS/NKX6.1, CHGA/MAFA, and INS/GCG double positive cells (n = 3). Data are expressed as means ± SD. (K) TEM images of INS granules in WT and MIR503HG‐KO SC‐β cells. Scale bars, 0.1 µm. L) Proportions of INS granule‐containing cells quantified by morphological analysis of TEM images of SC‐β cells (n = 8). Data presented as mean values ± SD. M) GSIS of WT and MIR503HG‐KO SC‐β cells. N–P) INS secretion after stimulation with tolbutamide (n = 5), exendin‐4 (n = 5), and KCl (n = 5). L) qRT‐PCR analysis of pancreatic developmental and maturation marker expressions for WT and MIR503HG‐KO SC‐β cells. Q) IF for C‐peptide/KI67 of WT and MIR503HG‐KO SC‐β cells. Scale bars, 20 µm. These results were repeated in at least three independent differentiations. All data are expressed as means ± SD. Statistical significance was calculated using a two‐tailed Student's *t*‐test.

Next, we investigated whether MIR503HG KO in hESCs would improve SC‐β cell maturation and function when entering the maturation stage of differentiation. MIR503HG^−/−^ PP cells aggregated into islet‐like clusters similar to WT PP cells in brightfield (**Figure** **3**A). However, based on the immunostainings, significantly more cells co‐expressed INS/Glucagon (GCG) and PDX1/NKX6.1 in the early formation (7 days) of MIR503HG^−/−^ SC‐β cells (Figure 3B). On day 21 of stage 6, SC‐β cells clusters were digested into single cells, then cultured in flasks for 2D culture (Figure 3C). The IF staining revealed robust nuclear MAFA expression and cytoplasmic C‐peptide expression in the NKX6.1^+^ population of MIR503HG^−/−^ SC‐β cells, compared to that in WT cells (Figure 3D).

Additionally, the FCM results demonstrated that the percentage of INS and NKX6.1 double‐positive cells were significantly increased in MIR503HG‐KO cells (49.60 ± 4.00% vs 77.37 ± 3.20%; Figure 3E,H). Many more KO cells co‐expressed endocrine cells induction marker CHGA and the maturation marker MAFA increased in KO cells (34.43 ± 2.90 vs 45.4 ± 2.10%; Figure 3F,I). Conversely, MIR503HG KO reduced the proportion of INS^+^/GCG^+^ ploy‐hormonal cells with respect to control cells (14.43 ± 2.01 vs 8.97 ± 1.17%; Figure 3G,J).

Mature pancreatic β cells can reportedly respond to glucose stimulation and secreteINS.^[^ ^3b^ ^]^ Our TEM images revealed that MIR503HG^−/−^ SC‐β cells contained more typical INS granules, suggesting a better INS‐producing capacity (Figure 3K,L). We measured INS secretion using several classical stimulants (glucose, tolbutamide, exendin‐4, and KCl) and concluded that it raised moderately in MIR503HG^−/−^ SC‐β cells (Figure 3M–P). β cell functional maturation is reportedly associated with reduced proliferation.^[^ ^23^ ^]^ MIR503HG^−/−^ SC‐β cells exhibited more C‐peptide and less KI67 expression and displayed altitudinal maturation and safety (Figure 3Q; Figure S5A,B, Supporting Information). However, we examined the pancreatic markers expression of WT and KO SC‐β cells and found that MIR503HG depletion increased the expression levels of pancreatic lineage‐crucial TFs PDX1 and NKX6.1, maturation marker MAFA, and endocrine markers INS and GCG. Remarkably, the δ cell marker SST expression levels decreased in MIR503HG^−/−^ SC‐β cells. Otherwise, the expression levels of pancreatic islet marker ISL expression levels were identical in the two KO and WT cell lines (Figure S5C,D, Supporting Information). Taken together, these results demonstrate that MIR503HG^−/−^ SC‐β cells displayed improved functional maturation.

### 3.4 MIR503HG^−/−^ Cells could Rapidly Reverse Hyperglycemia In Vivo

**Figure 4:**
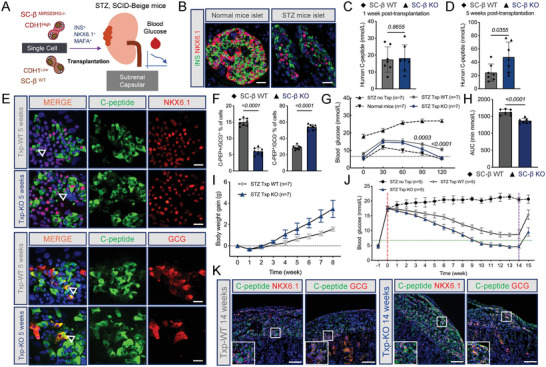
MIR503HG−/− cells could rapidly reversed hyperglycemia in vivo. A) Schematic diagram for in vivo functional assays of transplanted SC‐β cells. B) IF for INS/NKX6.1‐double positive normal or STZ‐induced diabetic mouse islets. Scale bars, 50 µm. C,D) Human C‐Peptide content in the serum of mice grafted with WT and MIR503HG‐KO β cells 1 week or 5 weeks post‐transplantation. E) IF for C‐Peptide/NKX6.1 and C‐Peptide/GCG of grafts from mice transplanted with WT and MIR503HG‐KO β cells 1 week or 5 weeks post‐transplantation. Scale bars, 50 µm. F) Calculated percentages for C‐PEP^+^/GCG^+^ or C‐PEP^+^/GCG^−^ positive of WT and MIR503HG‐KO groups by morphological analysis of IF images of SC‐β cells (n = 8) using ImageJ. Data presented as mean values ± SD. G) IPGTT analysis of mice transplanted with different SC‐β cells and without transplantation (n = 7). H) AUC analysis of mice transplanted with different SC‐β cells (n = 7). I) Body weight gain of mice with different SC‐β cells (n = 7). J) Blood glucose of mice transplanted with different SC‐β cells and without transplantation for several weeks (n = 7). K) IF for C‐Peptide/NKX6.1 and C‐Peptide/GCG of grafts from mice transplanted with KO and WT β cells 14 weeks post‐transplantation. Scale bars, 100 µm. These results were repeated in at least three independent differentiations. All data are expressed as means ± SD. Statistical significance was calculated using a two‐tailed Student's *t*‐test.

To test the in vivo efficacy of MIR503HG^−/−^ SC‐β cells, we transplanted 3‐million‐cell‐containing WT or MIR503HG^−/−^ SC‐β cell clusters into the kidney capsules in diabetic mice with streptozotocin (STZ)‐induced INS‐dependent diabetes mellitus (**Figure** **4**A,B). No significant difference in the human C‐peptide serum levels was observed between WT or MIR503HG^−/−^ SC‐β transplanted mice 1 week after transplantation (Figure 4C), whereas the KO cells produced nearly two times human C‐peptide content than that of WT cells at 5 weeks (Figure 4D). We immunostained and analyzed grafts under the kidney capsule 5 weeks post‐transplantation and observed that more C‐peptide/NKX6.1 and less C‐peptide/GCG‐double positive cells were present in the MIR503HG^−/−^ than those in the WT SC‐β cell grafts (Figure 4E). Further analysis for IF revealed that MIR503HG^−/−^ SC‐β cell grafts were mature for more mono‐hormonal C‐peptide^+^ (C‐PEP^+^)/GCG^−^ cells (53.96 ± 3.28 vs 28.76 ± 2.54%) and less poly‐hormonal C‐PEP^+^/GCG^+^ cells (6.02 ± 1.10 vs 14.99 ± 1.10%) generated in KO groups compared to those in WT groups (Figure 4F).

Next, we verified and measured the body weight gain, blood glucose levels as well as glucose tolerance and clearance functions. The blood glucose level changes during the 120 min after the intraperitoneal glucose injection revealed that diabetic mice carrying MIR503HG^−/−^ SC‐β cells exhibited significantly better glucose tolerance and clearance compared to those carrying WT SC‐β cells (Figure 4G,H). Mice transplanted with MIR503HG^−/−^ SC‐β cells gained a significantly increased amount of body weight after transplantation compared to mice grafted with WT SC‐β cells at 8 weeks (Figure 4I). Accordingly, diabetic mice with MIR503HG^−/−^ SC‐β cells and nondiabetic untreated mice regulated blood glucose homeostasis similarly; the blood glucose levels of the former displayed marked decline, recovered to normal at 9 weeks, and remained stable within our observation period. Notably, WT SC‐β cells could not regulate blood glucose levels of the recipients as normal. Additionally, soon after the removal of the grafts, recipients of both groups suffered from hyperglycemia, indicating the effects on glucose homeostasis of transplanted SC‐β cells (Figure 4J). Next, we performed immunostaining and analyzed the grafts under the kidney capsule 14 weeks after transplantation. Compared to the WT group, more C‐peptide^+^/NKX6.1^+^ SC‐β cells and less C‐peptide^+^/GCG^+^ hormone cells exited in MIR503HG^−/−^ grafts, indicating that MIR503HG KO promoted mono‐hormone‐producing cells formation in vivo with β cell‐specific characteristics (Figure 4K). In addition, a higher proportion of INS^+^ cells were observed in MIR503HG^−/−^ grafts compared to the KI67^+^ cells (Figure S6A, Supporting Information). Furthermore, INS did not co‐express with KI67 in the SC‐β cells, demonstrating that proliferative cells were immature in INS production (Figure S6B, Supporting Information). After 15 weeks post‐transplantation, much less KI67^+^ cells were detected in MIR503HG^−/−^ grafts compared to WT grafts (Figure S6C, Supporting Information). Macroscopic and microscopic examinations of the major organs revealed no evidence of post‐transplant tumorigenesis (Figure S6D, Supporting Information). In a word, these results suggest that MIR503HG^−/−^ SC‐β cells can improve hyperglycemia efficiently, quickly, and safely during the observation period to some extent.

### 3.5 Loss of MIR503HG Improved Pancreatic Lineage Specification

As pancreatic lineage specification is fundamental for β cell differentiation and maturation, we next focused on clarifying how MIR503HG KO can affect specification. Bright images revealed that in the DE, PG, PFG, and PP stages, MIR503HG**^−/−^** cells were significantly more homogeneous in size, morphology, and shape compared to those in WT cells (Figure S7A, Supporting Information). We then performed mRNA‐seq to assess the possible transcriptome‐related changes during the development of the two groups. Gene ontology (GO) and Kyoto Encyclopedia of Genes and Genomes (KEGG) analyses showed that terms enriched among DEGs and pathways were related to development in the DE and PG along with about development and adhesion in the PFG and PP. Several DE, PG, PFG, and PP stage marker genes were transcriptionally upregulated after MIR503HG loss. qRT‐PCR analysis confirmed that MIR503HG KO significantly increased the expression of PP marker genes, including PDX1, NKX6.1, SOX9, and other crucial TFs (Figure S7B, Supporting Information). Notably, the loss of MIR503HG increased CDH1 mRNA transcription when cells entered the PFG and PP stages, suggesting a regulatory role (Figure S7C–F, Supporting Information). Consistent with the qRT‐PCR results, mRNA‐seq data demonstrated that MIR503HG deficiency facilitated the transcription of some key PP TFs (Figure S7F, Supporting Information). Specification for the fate of endoderm‐derived organs follows the back of the formation of gastrulation, such as the liver and pancreas, whereas progenitors are thought to originate from the anterior definitive endoderm in other studies.^[^ ^24^ ^]^ Consequently, we investigated whether MIR503HG was a specific regulator of pancreatic lineage allocation. A planar differentiation protocol was adapted to obtain stem cell‐derived hepatocytes (SC‐hepatocytes) to test the liver potency (Figure S8A, Supporting Information). IF was performed to examine the expression levels of hepatocyte markers, and the data showed that SC‐hepatocytes of the two groups co‐expressed ALB/HNF4A equally (Figure S8B,C, Supporting Information). The expression of some characteristic marker genes was confirmed, with no significant difference between KO and WT cells containing HNF4A, ALB, CYP3A4, CK19, and LGR5 (Figure S8D, Supporting Information). In addition, MIR503HG KO did not have influence on CK19/HNF4A co‐expression, whereas polyploidy cells were observed in both groups as a feature for mature hepatocytes. Furthermore, ELISA for ALB and urea showed that MIR503HG KO did not affect the secretion and metabolism of hepatocytes (Figure S8E–G, Supporting Information). In conclusion, these results demonstrate that MIR503HG loss does not affect liver fate specification of SC‐hepatocytes.

**Figure 5:**
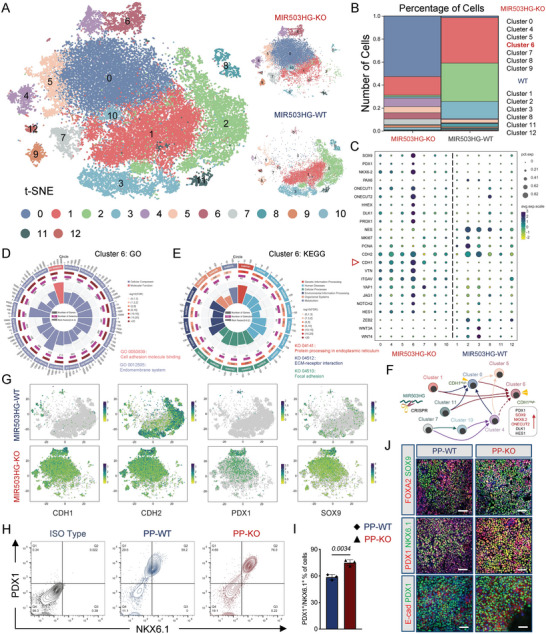
Loss of MIR503HG improved pancreatic lineage specification. A) *t*‐SNE projection from unsupervised clustering of transcriptional data from WT and MIR503HG‐KO PFG scRNA‐seq. B) Calculated percentage of defined WT and MIR503HG‐KO PFG clusters. C) Dot plot presenting DEGs in different cell clusters. Color and size of each dot represent expression levels and the percentage of cells expressing the given gene, respectively. D) GO and E) KEGG analysis for DEGs between WT and MIR503HG‐KO PFGs. F) Representative chart of CDH1^high^ and CDH1^low^ cell clusters defined by scRNA‐seq. G) *t*‐SNE projection of cells colored based on the expression of CDH1, CDH2, PDX1, and SOX9. H) FCM of WT and MIR503HG‐KO PPs for PDX1/NKX6.1. I) Population percentage of PDX1/NKX6.1 double positive cells (n = 3). Data are expressed as means ± SD. J) IF for FOXA2/SOX9, PDX1/NKX6.1, and E‐cad/PDX1 of WT and WT and MIR503HG‐KO PPs. Scale bars, 50 µm. FCM and staining results were repeated in at least three independent differentiations. All data are expressed as means ± SD. Statistical significance was calculated using a two‐tailed Student's *t*‐test.

To address how MIR503HG affects intermediate processes during SC‐β cells development at the cell cluster level, we performed scRNA‐seq on day 2 when PFG converted into PP cells. All cells were divided into 13 clusters using *t*‐SNE (**Figure** **5**A); the main clusters of KO cells were Clusters 0, 4, 5, and 6 whereas Clusters 1, 2, 3, and 8 were mainly in the WT group (Figure 5B). Next, we plotted several PP development‐related genes both from the WT and MIR503HG^−/−^ cells. PP specification genes, such as PDX1, SOX9, NKX6.1, NKX6.2, GATA6, and ONECUT1/2, and cell adhesion or junction‐related genes, such as CDH1, VTN, ITGAV, and LAMA1, were significantly increased in Clusters 0, 4, 5, and 6. Mesenchymal markers NES and ZEB1/2 were upregulated in Clusters 1, 2, 3, and 8 of the WT cells. Interestingly, another main cadherin‐encoding mRNA CDH2 was equally enriched in nearly all clusters (Figure 5C). We then carried out the RNA velocity estimation and pseudotime analysis of the MIR503HG‐KO subpopulations to infer a differentiation trajectory to investigate the appearance and transformation of each cluster (Figure S9A–D, Supporting Information). Cluster 6 cells were most likely the end point along the differentiation trajectory, in which there was a high expression of key TFs, including PDX1, NKX6.2, and SOX9 (Figure 5F). As the largest cluster of WT cells, Cluster 1 cells began to transform into Cluster 0 cells on the second day after differentiation of PFGs into PPs. Cluster 6 exhibited the highest number of ligand‐receptor pairs and increased communication with other clusters, suggesting a central position during pancreatic cell fate choice in this in vitro differentiation (Figure S9E, Supporting Information). Moreover, based on GO enrichment analysis, cell and biological adhesion as well as cadherin binding were among the top 20 enriched biological processes in Cluster 6 (Figure 5D). KEGG analysis identified high expressed genes of Cluster 6 were enriched in ECM‐receptor interaction, focal adhesion, and cell adhesion signaling pathways (Figure 5E). As a bulk sample, the expression of TFs for pancreatic development increased in KO cells compared to that in WT cells (Figure S9F, Supporting Information). GO and KEGG analysis revealed the inactivated disease‐associated pathways and cell cycle pathway, whereas adhesion ‐associated pathways were activated (Figure S9G,H, Supporting Information). Further analysis revealed an increase in noncycling cells and a significant reduction in G1 cells in MIR503HG^−/−^ PPs, implying cell cycle suppression upon MI503HG KO (Figure S9I,J, Supporting Information). Moreover, based on the cell cycle analysis, the noncycling cells were mainly distributed in Cluster 6, suggesting a relatively quiescent state in this cluster. MIR503HG loss has been shown to accelerate the transformation of PFGs to PPs with less time consumption.

Transformation of Cluster 1 into Cluster 0 was followed by eventual generation of Cluster 6 with high CDH1 expression observed along PP differentiation avenue (Figure 5F). *t*‐SNE was performed to analyze genes expression and clusters distribution of CDH1, CDH2, PDX1, and SOX9. In contrast, PDX1 and SOX9 were enriched in the CDH1 positive clusters broadly (Figure 5G). We performed a series of experiments to validate the scRNA‐seq results. The FCM data indicated that the PDX1 and NKX6.1‐double positive ratio of MIR503HG‐KO‐derived PP cells was much higher than that of WT‐derived PP cells (74.93 ± 3.33 vs 58.6 ± 3.09%; Figure 5H,I). qRT‐PCR results confirmed that CDH1, PDX1, and SOX9 mRNA expressions were upregulated, whereas that of MKI67 mRNA expression was downregulated, and CDH2 mRNA expression levels displayed no obvious differences, consistent with the sequencing results (Figure S10A, Supporting Information). We assessed the expression levels of several TFs and transmembrane cell‐cell adhesion molecules in both MIR503HG and WT PPs using IF. MIR503HG KD increased the FOXA2/SOX9 and PDX1/NKX6.1‐double positive cell proportions. Furthermore, PDX1‐positive PPs co‐expressed higher levels of E‐cad (Figure 5J). As differentiation proceeded to EP, upregulated expression of some endocrine TFs such as NGN3 and NKX6.1 as well as the pancreatic β cell TF PAX4 was observed in KO cells compared to that in WT cells, which was also confirmed using qRT‐PCR (Figure S10B–D, Supporting Information). In summary, these data suggest that MIR503HG loss accelerates the formation of both PPs and EPs.

### 3.6 MIR503HG Physically Interacted with CtBP1 in PPs

**Figure 6:**
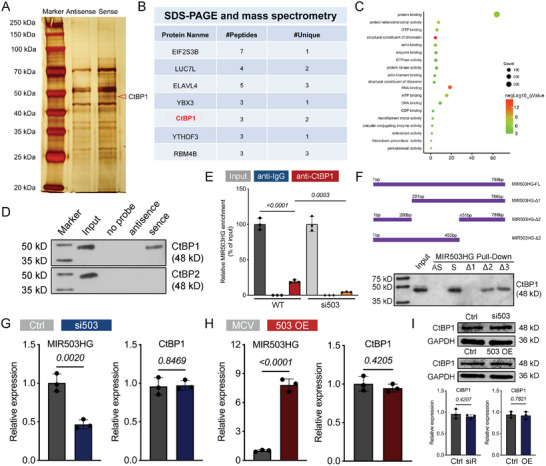
MIR503HG physically interacted with CtBP1 in PPs. A) Silver staining of RNA pull‐down proteins for MIR503HG. Highlighted region was cut and submitted for subsequent mass spectrometry. Arrowhead indicates CtBP1 at bands between 40–50kDa. B) List of proteins detected by mass spectrometry. C) GO analysis of MIR503HG‐enriched proteins after RNA pull down. D) WB analysis for the specific interaction between MIR503HG and CtBP1. E) RIP enrichment and qRT‐PCR analyses for determining whether MIR503HG was associated with CtBP1 relative to the input control. F) Deletion mapping of CtBP1‐binding domain in MIR503HG using FL (full length)‐AS as a negative control. G,H) qRT‐PCR analysis of MIR503HG and CtBP1 expression level for PPs after MIR503HG knockdown or overexpression. I) WB analysis for CtBP1 protein after MIR503HG KD or overexpression using GAPDH as a loading control. Relative optical density analyzed by ImageJ. Results were repeated in at least three independent differentiations. All data are expressed as means ± SD. Statistical significance was calculated using a two‐tailed Student's *t*‐test.

LncRNAs often participate in molecular regulation via their interactions with chromatin‐modifying complexes or heterogeneous nuclear ribonucleoproteins.^[^ ^25^ ^]^ To explore the function of MIR503HG could function during pancreatic specification, we carried out RNA‐protein pull‐down to identify an RNA‐binding protein (RBP) for MIR503HG by incubating transcribed biotinylated MIR503HG in vitro and an antisense RNA with PP extracts. We detected a specific protein band at ≈ 48–50 kDa in the MIR503HG pull‐down samples among which proteins were enriched in protein binding (**Figure** **6**A,C), and identified 7 potential interacting proteins by mass spectrometry (Figure 6B). Based on the peptide binding rate, ELAVL4 and CtBP1, among 7 potential RBPs reported in some studies on stem cells and differentiation, were chosen for further mechanistic study. The CtBP1 and CtBP2 isoforms constitute the CtBP family in mammalians with diverse functions in embryogenesis and development.^[^ ^26^ ^]^ CtBP1 selective binding to MIR503HG was further confirmed by western blot (WB) analysis (Figure 6D). However, ELAVL4 did not appear to bind to MIR503HG by WB (data not shown). In contrast, MIR503HG was significantly enriched in CtBP1 immunoprecipitates but not in other controls (Figure 6E). These results indicate that LINC MIR503HG specifically interacts with CtBP1 but not with CtBP2 in PPs. To further map the specific CtBP1 binding region, we constructed 3 MIR503HG deletion mutants based on the predicted secondary structure using RNAfold (Figure S11A, Supporting Information, Figure 6F). In addition, miR‐503 and miR‐424 were located at the MIR503HG locus (Figure S11B, Supporting Information). However, the pancreatic β cell TF transcript levels were indistinguishable between the two miRNAs inhibition and relative control groups (Figure S11C,D, Supporting Information), suggesting that MIR503HG partial sequence disturbance did not influence PP differentiation. In other words, the acceleration in the PP cell fate decision was counted on the KD or KO of the entire MIR503HG sequence. The RNA pulldown and WB analyses identified the common MIR503HG‐△1–2 (0—200 bp) as an essential CtBP1‐binding region (Figure 6F; Figure S12A–C, Supporting Information). Neither MIR503HG KD nor overexpression affected CtBP1 transcription or translation (Figure 6G–I). RBP CtBP1 binding‐mediated MIR503HG function in PP cell fate designation prompted us to perform further studies. Based on these results, we hypothesized that MIR503HG is involved in pancreatic lineage specification via a physical interaction‐based interplay with CtBP1.

### 3.7 MIR503HG was Indispensable for CtBP1 in the Transcriptional Co‐Repression of E‐Cad and HES1 in PPs

**Figure 7:**
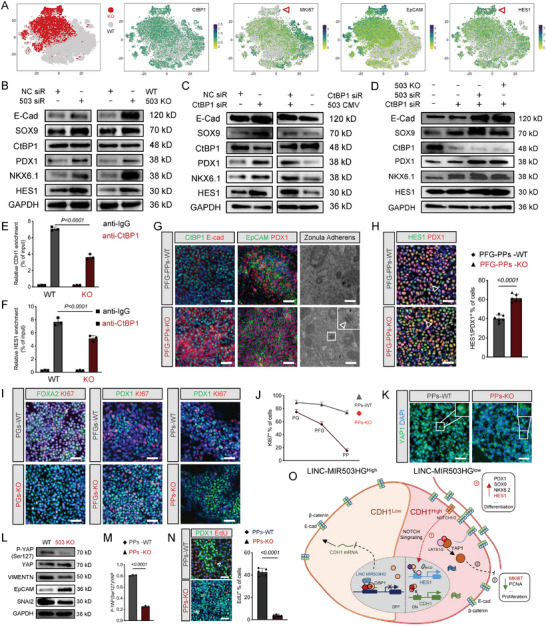
MIR503HG was indispensable for CtBP1 in the transcriptional co‐repression of E‐cadherin and HES1 in PPs. A) *t*‐SNE projection of WT and MIR503HG‐KO PPs colored based on the expression of CtBP1, MKI67, EpCAM, and HES1. Arrowhead denotes Cluster 6. B–D) WB analysis highlighting changes in E‐cad, PDX1, NKX6.1, CtBP1, SOX9, and HES1 of different groups using GAPDH as a loading control. E,F) Chip‐qRT‐PCR analysis for CtBP1 occupation of CDH1 mRNA and HES1 mRNA promoters. G) IF for CtBP1/E‐cad and EpCAM/PDX1 of WT and MIR503HG‐KO PPs. Scale bars, 50 µm. TEM images of Zonula Adherens in WT and MIR503HG‐KO SC‐β cells. Scale bars, 0.1 µm. Arrowhead indicates zonula adherens. H) IF for HES1/PDX1 of WT and MIR503HG‐KO PPs. Scale bars, 50 µm. Calculated percentages for HES1 and PDX1 positive of WT and MIR503HG‐KO groups by morphological analysis of IF images of SC‐β cells (n = 6) using ImageJ. Data presented as mean values ± SD. I) IF for FOXA2/KI67 and PDX1/KI67 of WT and MIR503HG‐KO PGs, PFG, and PPs separately. Scale bars, 50 µm. J) Calculated percentage for KI67‐positive WT and MIR503HG‐KO PGs, PFG, and PPs by morphological analysis of IF images of SC‐β cells (n = 6) using ImageJ. Data presented as mean values ± SD. K) IF for YAP1 of WT and MIR503HG‐KO PPs. Scale bars, 50 µm. L) WB analysis for p‐YAP1, YAP1, VIMENTIN, EpCAM, and SNAI2 of WT and MIR503HG‐KO PPs using GAPDH as a loading control. M) Calculated percentage for p‐YAP/YAP1 ratio of WT and MIR503HG‐KO PPs. Relative optical density analyzed by ImageJ. Experiments were performed in triplicate. N) IF for PDX1/EDU‐double positive of WT and MIR503HG‐KO PPs. Scale bars, 50 µm. Calculated percentage for EdU positive of WT and MIR503HG‐KO PPs by morphological analysis of IF images of SC‐β cells (n = 5) using ImageJ. Data presented as mean values ± SD. O) Schematic diagram for a proposed model of MIR503HG function during PP differentiation and proliferation. Results were repeated in at least three independent differentiations. All data are expressed as means ± SD. Statistical significance was calculated using a two‐tailed Student's *t*‐test.

Several CtBP transcriptional co‐repressor complexes are reportedly involved in TF gene silencing during various developmental processes, mainly in malignant tumors.^[^ ^27^ ^]^ Moreover, CtBP1, MKI67, HES1, and EpCAM were mapped to the *t*‐SNE. CtBP1 distribution was uniform between the two groups, whereas HES1 and EpCAM were highly expressed in MIR503HG^−/−^ PP subpopulations but MKI67 was not (**Figure** **7**A). To decipher how the combination of MIR503HG and CtBP1 affects PPs, we focused on the role of MIR503HG in pancreatic cell fate determination. At the onset of pancreatic branching, E‐cad is highly expressed in body cells and is thought to differentiate into endocrine or ductal cells in vivo.^[^ ^28^ ^]^ IF and WB analyses revealed that MIR503HG KO resulted in significantly higher E‐cad, EpCAM, and PDX1 without disturbance CtBP1 (Figure 7B,G,L). Furthermore, epithelial phenotype marker expression levels were significantly higher in MIR503HG^−/−^ PPs compared to those of the mesenchymal phenotype (Figure 7L). HES1, as a Notch pathway target, promotes proliferation and maintains high PP multipotency.^[^ ^29^ ^]^ Higher PDX1‐ and HES1‐coexpressing PP ratios were observed in the MIR503HG KO compared to those in the WT group (Figure 7H). These results suggested that MIR503HG^−/−^ facilitated pancreatic branching and PP multipotency.

Next, we focused on the mechanism underlying the loss of MIR503HG in PPs. MIR503HG KD led to the downregulation of PDX1, NKX6.1, and SOX9, which are crucial for PPs (giving rise to all adult pancreatic endoderm cells), and was enhanced by MIR503HG depletion, as expected (Figure 7B). Recent studies have confirmed that CtBP1 represses CDH1 and HES1 transcription by binding to their promoters during developmental processes.^[^ ^20^, ^30^ ^]^ Our chip‐PCR results identified E‐cad and HES1 promoters as potential CtBP1 targets in this modified differentiation model (Figure 7E,F). Similarly, PDX1, NKX6.1, and SOX9 expression levels increased in PPs transfected with CtBP1 siRNA targeting E‐CAD and HES1 repression (Figure 7C). In contrast, MIR503HG overexpression did not reinforce this inhibition, which was consistent with data of qRT‐PCR (Figure 6H and 7C). Co‐transfection of MIR503HG and CtBP1 siRNA combined with further MIR503HG loss in PPs, strengthened the release of E‐cad and HES1 (Figure 7D). Thus, we speculated that MIR503HG may be indispensable for the CtBP1‐mediated gene silencing.

As differentiation progressed toward the PP cell‐fate decision, the proliferative ability sharply declined in MIR503HG^−/−^ cells compared to high and stable proliferation in WT cells (Figure 7I,J,N). Excessive PP proliferation hampers endocrine cell fate initiation by repressing Neurog3 expression, resulting in an exocrine ductal fate via CK19 activation. Hippo/YAP pathway inactivation reportedly results in the loss of PP proliferation, promoted by phosphorylated YAP (p‐YAP), as assayed by endogenous YAP subcellular localization and the p‐YAP/total YAP ratio.^[^ ^31^ ^]^ WB analysis showed that the p‐YAP/total YAP ratio was lower in MIR503HG^−/−^ PPs, which was consistent with IF results indicating that more p‐YAP localizes in the cytoplasm than does dephosphorylated YAP in the nucleus (Figure 7K–M), where it interacts with other TFs.^[^ ^32^ ^]^ PDX1/NKX6.1‐double positive MIR503HG^−/−^ PPs were over 80% with no proliferative capacity due to Notch and YAP pathway inactivation, suggesting a disproportionate progenitor balance between self‐renewal and differentiation with high transformation potentials into EPs (Figure 5H and Figure 7O).

## 4 Discussion

During pancreatic development, PPs with inherent proliferation as well as PDX1 and NKX6.1 co‐expression are responsible for the proper specification of pancreatic β cells.^[^ ^33^ ^]^ hESCs and hiPSCs can be differentiated into PPs, which can then give rise to functional β‐like cells both in vitro and in vivo.^[^ ^34^ ^]^ Therefore, promoting PP acquisition and quality is a promising approach, as it would allow for the use of fewer cells if they were more mature and of a higher grade, thereby alleviating current challenges (i.e., implanting large volumes and more mature cells) that would theoretically be improbable to differentiate into off‐target unwanted populations.

However, despite evidence indicating that lncRNAs are crucial for proper β cell development and function.^[^ ^35^ ^]^, a systematic assessment of the global lncRNA expression patterns and specific regulatory mechanisms during pancreas development have yet to be investigated. In this study, scRNA‐seq revealed that increased MIR503HG expression levels were accompanied by mesenchymal characteristics such as FN1, CDH2, VIM, SNAI2, and NES. Moreover, its deficiency results in the appearance of epithelial cells expressing EpCAM, CDH1, VTN, and LAMA1.^[^ ^36^ ^]^ Thus, MIR503HG loss promotes the fast mesenchymal‐epithelial transition. The embryonic pancreas, which gives rise to β cells, undergoes early epithelial rearrangements, including transient stratification of an initially monolayered epithelium, followed by microlumen formation and subsequent branching. Sustained EMT promotes β cell identity loss and impairs INS secretion via dedifferentiation during human islet expansion in vitro.^[^ ^36b,c^ ^]^.

As described above, MIR503HG was not indispensable or even adverse for β cell differentiation and maturation as the differentiation trajectory paced forward. In this study, data showed that MIR503HG KD or OK increased the expression of several pancreatic development‐related TFs both at the mRNA and protein levels. Notably, the MIR503HG loss promoted NKX6.2 but not NKX6.1, coordinating with PDX1, specifies the endocrine fate in PPs by inducing NGN3^+^ endocrine precursors, which then give rise to human pancreatic endocrine cells, including mainly β and α cells.^[^ ^37^ ^]^ Loss of Nkx6.1 even impedes pancreatic organogenesis.^[^ ^37a^ ^]^ Nkx6.2, another important Nkx6 factor, can induce Nkx6.1 downstream effectors and β cell‐specific markers.^[^ ^38^ ^]^ Despite their different biochemical functions during the development of other organs, these two Nkx6 factors possess equivalent biochemical activities during endocrine differentiation.^[^ ^38a^ ^]^ The PP pools were enlarged upon the loss of MIR503HG‐enhanced PDX1, NKX6.1, SOX9, and FOXA2 expression, indicating the amplification of the progenitor state. Interestingly, MIR503HG KO increased the PDX1/NKX6.1 double positive PPs rather than PDX1 positive cells. NKX6.1 positive PPs are confirmed to adopt endocrine cell‐fate choice.^[^ ^38b^ ^]^ Whether MIR503HG KO PPs arrived state for endocrine cells earlier than WT PPs and the mechanism beneath this phenomenon need more studies.

MIR503HG^−/−^ PPs can be more efficiently differentiated into functional and higher‐quality β cells, exhibiting better ability to respond to different INS secretion stimulations in vitro and maintaining blood glucose homeostasis in vivo. In addition, these endocrine cells exhibited higher expression levels of MAFA, an important maturation marker,^[^ ^39^ ^]^ mono‐hormonal expression, and epithelial characteristics. In summary, MIR503HG^−/−^ SC‐derived β cells were significantly more mature, closer to the permanent cell state, and exhibited better glucose homeostasis regulation without jeopardizing graft efficacy in response to glucose challenge during the experimental period in vivo.

The majority of MIR503HG^−/−^ β cells were INS mono‐hormonal with fewer INS/GCG poly‐hormonal cells in vivo, which were considered to ultimately convert into GCG mono‐hormonal cells. Pax6, a key component in regulating α cell differentiation GCG gene transcription, GCG synthesis and secretion, and glucolipotoxicity, was repressed by MIR503HG deficiency.^[^ ^40^ ^]^ Moreover, NeuroD1, which reportedly activates GCG transcription in combination with the transcription factor E47, may indirectly contributed to the effect of Pax6 on GCG gene transcription.^[^ ^15^, ^41^ ^]^ Additionally, Pax6 reportedly binds to the GCG promoter and activates its expression by interacting with cMaf and MafB directly.^[^ ^42^ ^]^ In the present study, GCG and NEUROD1 expression was upregulated by MIR503HG KO along with PAX6 downregulation, indicating that MIR503HG loss can compensate PAX6 reduction for its adverse effects on GCG and NEUROD1 expression, which was enhanced actually and later α cell differentiation.

Intriguingly, in our studies, the expression and distribution of epithelial characteristics as CDH1 was opposite to that of PAX6. We hypothesized whether mesenchymal characteristics would facilitate the expression of PAX6 and α cells development. As reported, α cells encircle main most β cells adjacently potentially due to β cell plasticity, involving cadherins and cytoskeletal proteins.^[^ ^43^ ^]^ A recent study demonstrated that intra‐islet GCG signaling is required for efficient INS release in vivo and proper regulation of blood glucose homeostasis.^[^ ^44^ ^]^ α‐β cell crosstalk is crucial for the cooperative function of healthy human α and β cells in Langerhans islets, as heterologous contacts between human β cells and α cells were also able to increase INS secretion.^[^ ^45^ ^]^ However, the specific crosstalk signals and subsequent regulatory mechanisms between these two major endocrine cell types remain to be elucidated.

Recent studies have highlighted the critical roles that RNA‐binding proteins (RBPs) can fulfill in modulating organogenesis, maturation, function, and cancer, alleviating endoplasmic reticulum stress by binding to RNAs and regulating their processing, stability, localization, modification, or translation.^[^ ^46^ ^]^ In the current study, MIR503HG, an intergenic lncRNA, repressed CDH1 and HES1 transcription by binding to the RBP, and CtBP1, and adversely affecting pancreatic lineage specification. Previously, CtBP1, a core component of the CTBP/ZEB/SNAIL/TWIST, a large nuclear transcriptional co‐repressor complex, as well as that of the CtBP1/LSD/CoRest complex and others, was recruited to promoter elements and acted as a bridge by interacting with DNA‐binding transcriptional repressors instead of direct DNA‐binding.^[^ ^47^ ^]^ Our data indicates that MIR503HG does not directly regulate CtBP1 transcription or translation, the loss of which markedly enhances the translation of certain crucial PP TFs. MIR503HG KD promotes PP differentiation and cell‐fate determination, which are further enhanced by simultaneous CtBP1 KD. However, CtBP1 KD did not enhance the promotion of MIR503HG loss during PP cell fate designation. We hypothesized that the transcriptional repression of CtBP1 relies on MIR503HG transcription. However, the mechanisms underlying this process require further investigation.

Cell fate determination relies on the precise regulation of several signaling pathways.^[^ ^48^ ^]^

In the present study, MIR503HG regulated PP cell fate via its participation in the repression of CtBP1 in the NOTCH signaling pathway downstream of the TFs HES1 and the adhesion molecule CDH1. During pancreatic development, PP proliferation and multipotency are maintained by the activation of Notch receptors.^[^ ^49^ ^]^ This activation results in the proteolytic cleavage of the Notch receptor followed by the release and nuclear translocation of the Notch intracellular domain (NICD) to activate HES1 expression, thereby inhibiting the master regulator of the endocrine fate Ngn3.^[^ ^50^ ^]^ We observed that the progenitor state was enhanced and PP quantities increased upon MIR503HG KO of upregulated Notch receptors including Jag1, Notch2, and HES1. Interestingly, as differentiation progressed, MIR503HG^−/−^ PPs were smoothly committed to the endocrine fate, bypassing prolonged influence from the NOTCH pathway, avoiding progenitor state retention, and eventually exocrine lineage fate.^[^ ^51^ ^]^ Notch inhibitor (γ‐secretase inhibitor Xxi) application in the EP stage conditional medium counteracted the effects of MIR503HG KO on PP station lingerer.^[^ ^52^ ^]^ Whether the DAPT dosage is optimal for the interplay between MIR503HG KO and the specific mechanism of action requires further investigation.

The Hippo/YAP pathway is another important signaling pathway, which participates in dominating the generation of PP‐derived functional β cells.^[^ ^53^ ^]^ The Hippo pathway reportedly integrates the tissue architecture by balancing progenitor cell self‐renewal and differentiation. Inhibition of Hippo signaling leads to nuclear translocation of the downstream effectors YAP and TAZ, which bind to TEAD coactivators and activate the transcription of genes controlling progenitor cell proliferation.^[^ ^54^ ^]^ In contrast, activation of the pathway promotes terminal differentiation of mature cell types by inducing the phosphorylation, cytoplasmic retention, and subsequent degradation of YAP/TAZ, resulting in target gene downregulation.^[^ ^55^ ^]^ Moreover, E‐cad is reportedly involved in cell growth inhibition by inactivating the Hippo pathway.^[^ ^56^ ^]^ In the present study, E‐cad expression was enhanced by the alleviation from the repression of binding between MIR503HG and CtBP1 in MIR503HG^−/−^ cells, resulting in PP proliferation constrained with increased phosphorylation and cytoplasmic YAP retention.

Taken together, our study demonstrates that MIR503HG is a promising target for SC‐β cell therapy. Accordingly, MIR503HG loss promotes PP differentiation, giving rise to higher‐quality SC‐β cells more efficiently and safely. Therefore, harnessing MIR503HG regulation will provide strategies for promoting human pancreatic differentiation and developing effective approaches for generating significant amounts of functional SC‐β cells for the fundamental study of pancreatic biology and regenerative medicine for diabetes.

## Conflict of Interest

The authors declare no conflict of interest.

## Author contributions

Y.X., S.M., H.F., and J.W. contributed equally to this work. Y.X., Z.J., and Y.H. conceived and designed the project. Y.X., H.F., S.M., and J.W. performed the experiments. Y.X., H.F., J.W., and Y.H. analyzed the sequencing data. M.Z., S.Z., Y.L. J.Y., and L.W. helped with the experiments. Y.X, J.W., S.M., and Y.H. analyzed the data. Z.W., Z.J., and Y.H. provided reagents. Y.X., H.F., J.W., B.Y., Z.W., Z.J., and Y.H. wrote and revised the manuscript.

## Supporting information

Supporting Information

## Data Availability

The data that support the findings of this study are available from the corresponding author upon reasonable request.
