# Supplementary material for: LINC MIR503HG Controls SC‐β Cell Differentiation and Insulin Production by Targeting CDH1 and HES1
Source: Adv Sci (Weinh). 2024 Jan 20;11(13):2305631. doi: 10.1002/advs.202305631 (PMC10987150; doi:10.1002/advs.202305631)
Supplement: Supplementary file 1 — Supporting Information [file ADVS-11-2305631-s001.pdf]

ADVANCED SCIENCE
Open Access

Supporting Information

for *Adv. Sci.*, DOI 10.1002/advs.202305631

LINC MIR503HG Controls SC-β Cell Differentiation and Insulin Production by Targeting CDH1 and HES1

Yang Xu, Susu Mao, Haowen Fan, Jian Wan, Lin Wang, Mingyu Zhang, Shajun Zhu, Jin Yuan, Yuhua Lu, Zhiwei Wang, Bin Yu, Zhaoyan Jiang and Yan Huang**

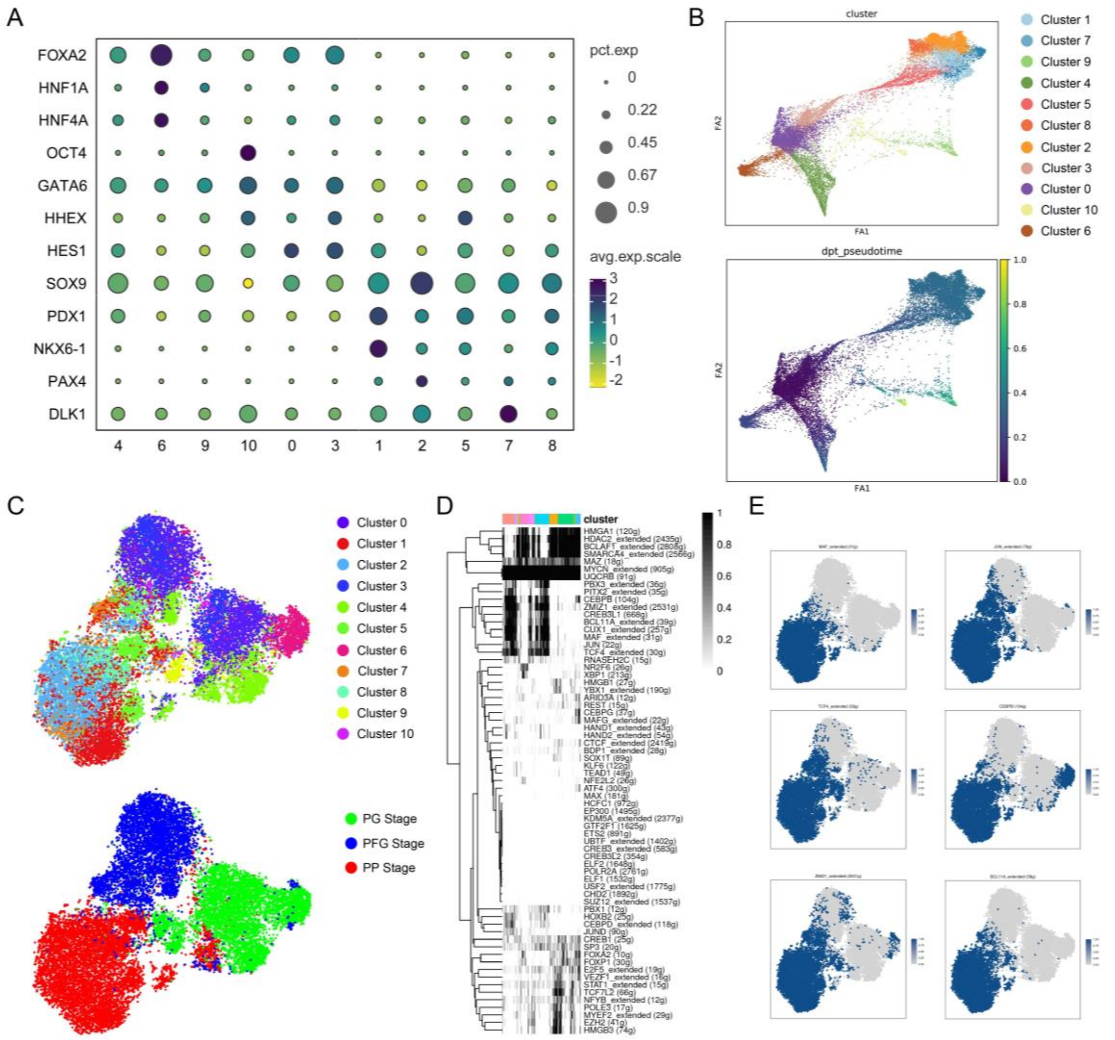

Figure S1. Additional analysis for PG, PFG, and PP scRNA-seq data. (A) Heatmap highlighting top common upregulated genes of PG, PFG, and PP cells. (B) Pseudotime distributions of PGs, PFGs, and PPs. (C) The *t*-SNE visualization of regulon openness in cells, colored by cell type. (D) Heatmap indicating TF regulon activities of different cell types. (E) *t*-SNE projection of cells colored based on expression levels of MAF, JUN, TCF4, CEBPB, ZMIZI, and BCL11A.

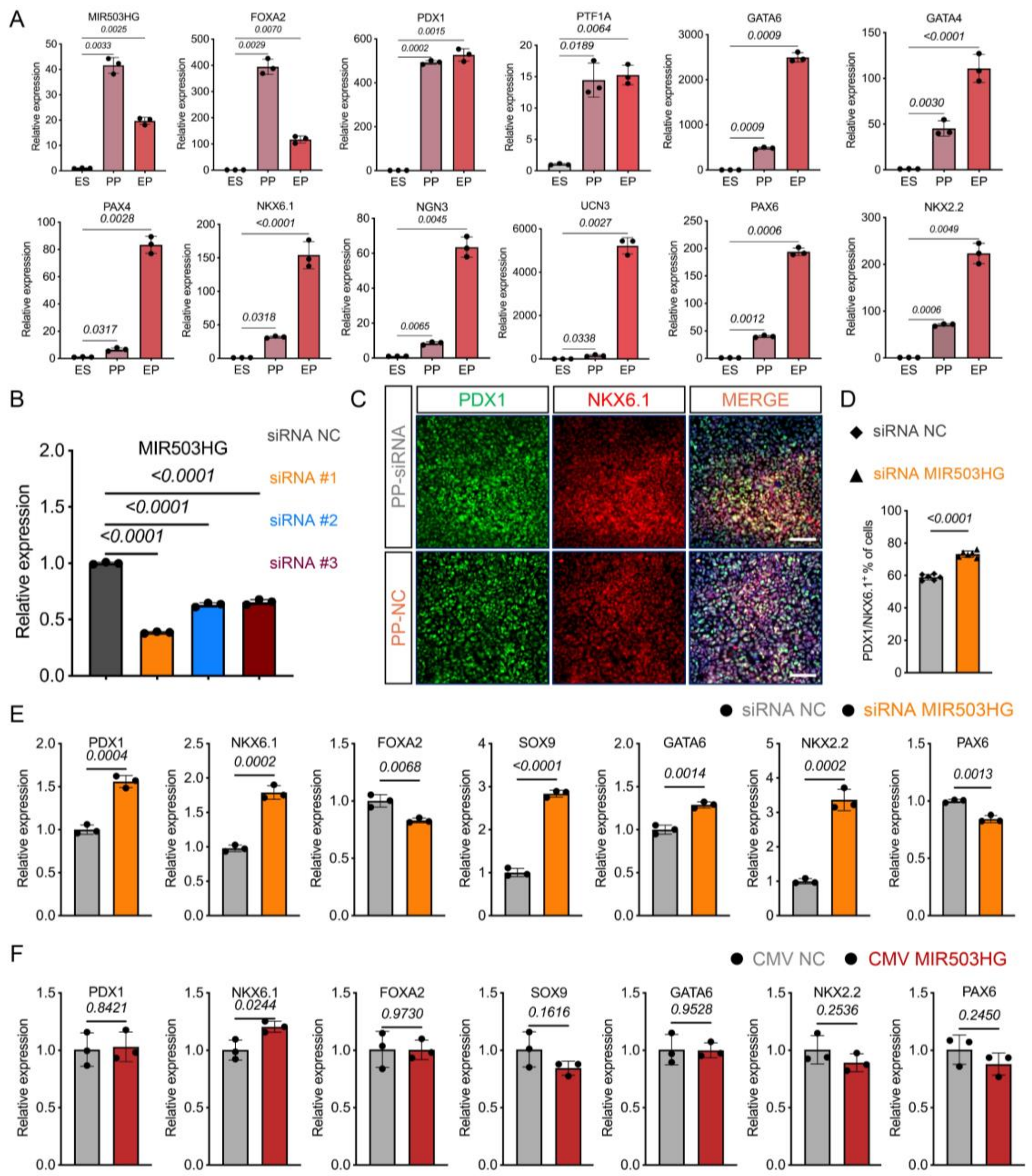

Figure S2. MIR503HG KD facilitated PP development whereas its overexpression had no influence.

(A) qRT-PCR analysis of MIR503HG, pancreatic developmental and maturation marker expression levels. (B) MIR503HG qRT-PCR expression levels for PPs transfected with different MIR503HG siRNA sequences. (C) IF for PDX1/NKX6.1 of PPs transfected with NC siRNA and MIR503HG siRNA #1. Scale bars, 50 μm. (D) Calculated percentage for PDX1/NKX6.1-double positive PPs transfected with NC siRNA or MIR503HG siRNA #1 by morphological analysis of IF images of SC-β cells (n = 6) using ImageJ. Data presented as mean values ± SD. (D and E) qRT-PCR analysis of pancreatic developmental and maturation marker expression levels for cells transfected with NC siRNA and MIR503HG siRNA #1 and NC CMV and MIR503HG CMV. Results were repeated in at least three independent differentiations. All data are expressed as means ± SD. Statistical significance was calculated using two-tailed Student's t-test.

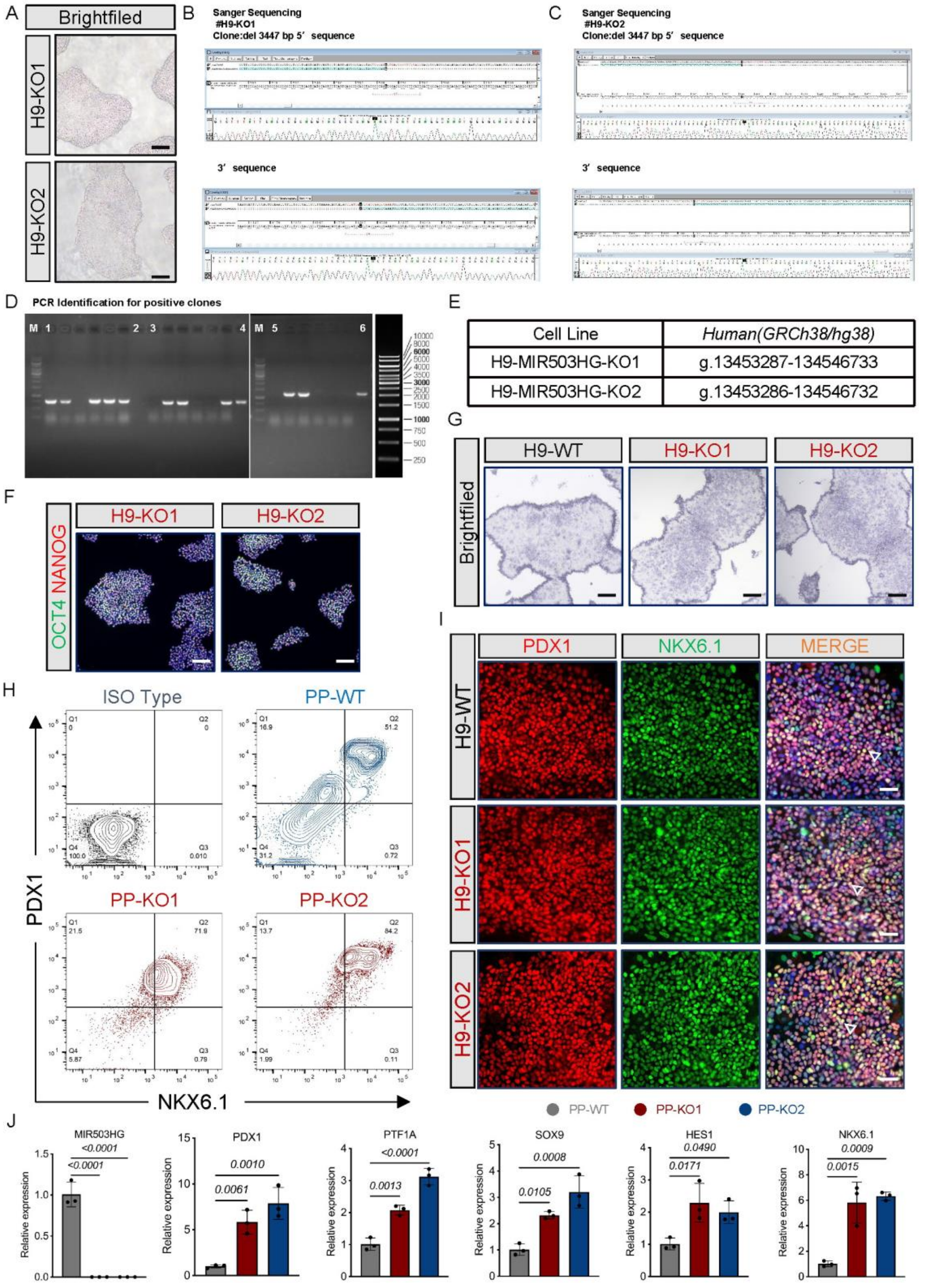

Figure S3. Identification of MIR503HG deletion. (A) Brightfield images of H9-MIR503HG-KO1 and KO2 ES cells. Scale bars, 500 μm. (B and C) Sanger sequencing of clone KO1 and KO2. (D) PCR identification of MIR503HG depletion in KO1. Lane 1: KO1, primer: F1/R1, fragment: 788bp; Lane 2: WT, primer: F1/R1, fragment: 4235bp; Lane 3: KO1, primer: F2/R1, fragment: 0bp; Lane 4: WT, primer: F2/R1, fragment: 701bp; Lane 5: KO1, primer: F1/R3, fragment: 0bp; Lane 6: WT, primer: F1/R3, fragment: 1204bp. (E) Information about two independent MIR503HG knockout (KO) hESC lines. (F) ALP staining

of WT, KO1, and KO2 ES cells. Scale bar, 500 μm. MIR503HG sequence for deletion. (G) IF for stem cell markers of KO1 and KO2 ES cells colonies. Scale bars, 500 μm. (H) FCM of WT KO1-PPs and KO2-PPs for PDX1/NKX6.1. Data were expressed as means ± SD. (I) IF for PDX1/NKX6.1 of WT-PPs, KO1-PPs and KO2-PPs. Scale bars, 50 μm. (J) qRT-PCR analysis of MIR503HG and pancreatic developmental marker expression levels. Results were repeated in at least three independent differentiations. All data are expressed as means ± SD. Statistical significance was calculated using two-tailed Student's t test.

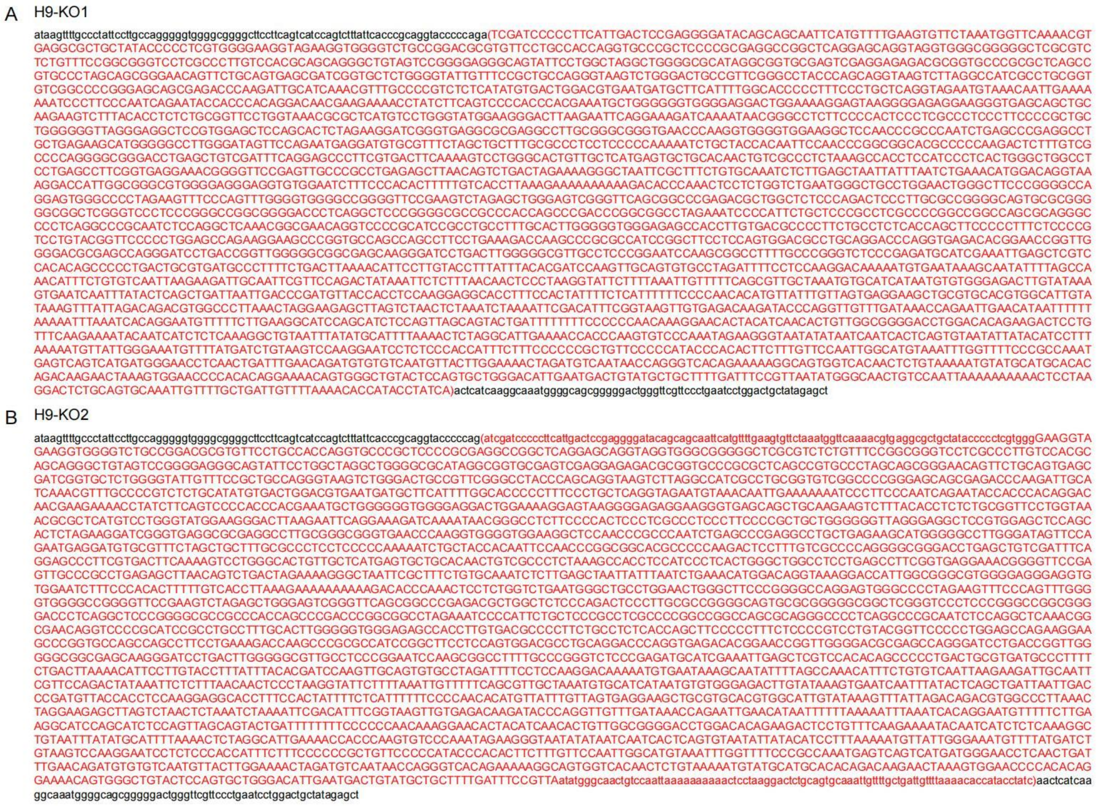

Figure S4. Sequences for MIR503HG depletion. (A and B) Sequences for MIR503HG depletion in KO1 and KO2 clones respectively. KO2 clones was chosen for following study.

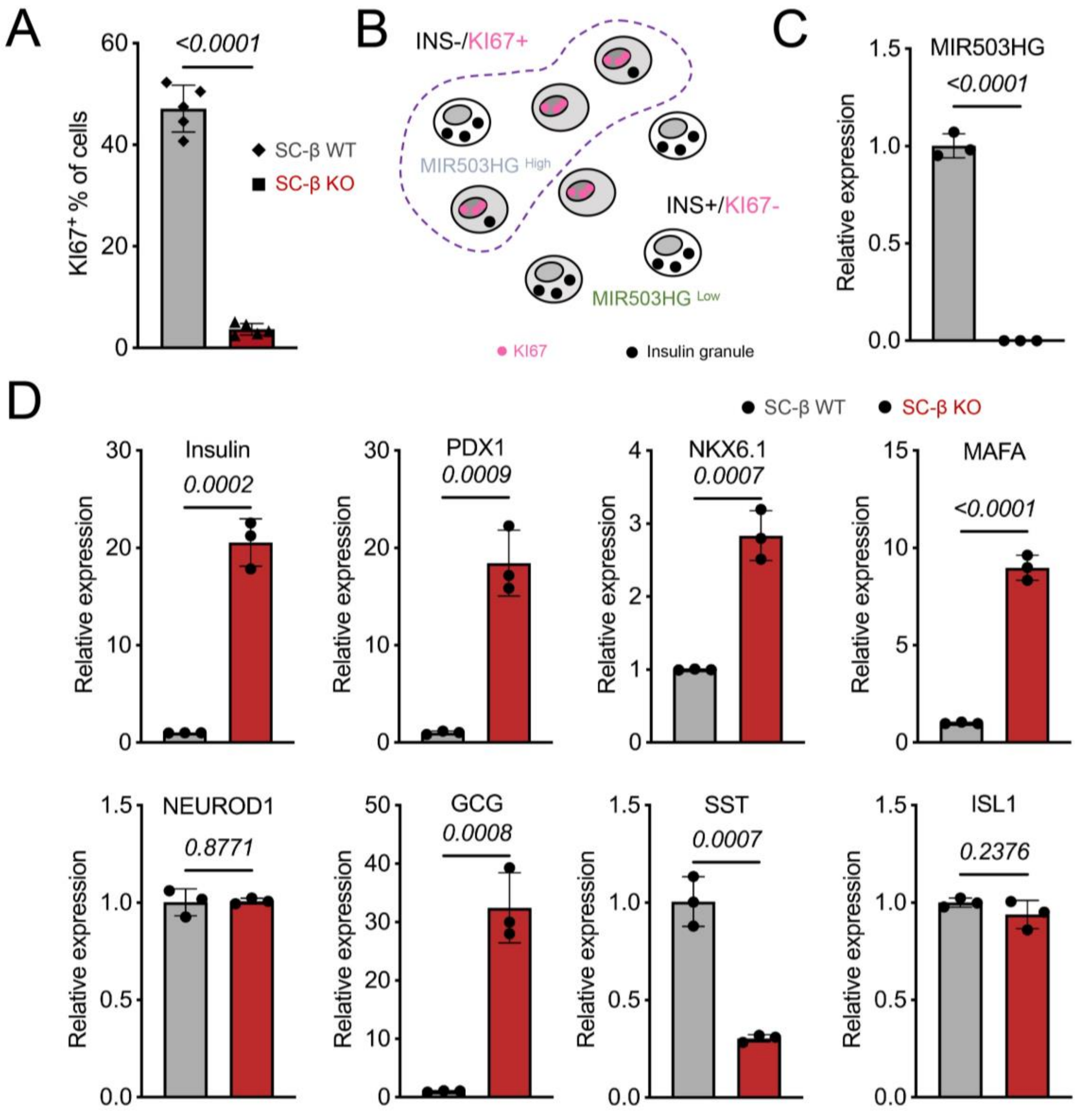

Figure S5. Additional analysis for WT and MIR503HG-KO SC-β cells. (A) Population percentage of KI67 positive cells quantified by morphological analysis of IF images (Fig. 3Q; n = 5). Data presented as mean values ± SD. (B) Schematic of SC-β cells with or without INS granule. (C) qRT-PCR analysis of the expression of MIR503HG for WT and MIR503HG-KO PPs. (D) qRT-PCR analysis of the expression of marker genes for WT and MIR503HG-KO PPs. Results were repeated in at least three independent differentiations. Statistical significance was calculated using two-tailed Student's t-test.

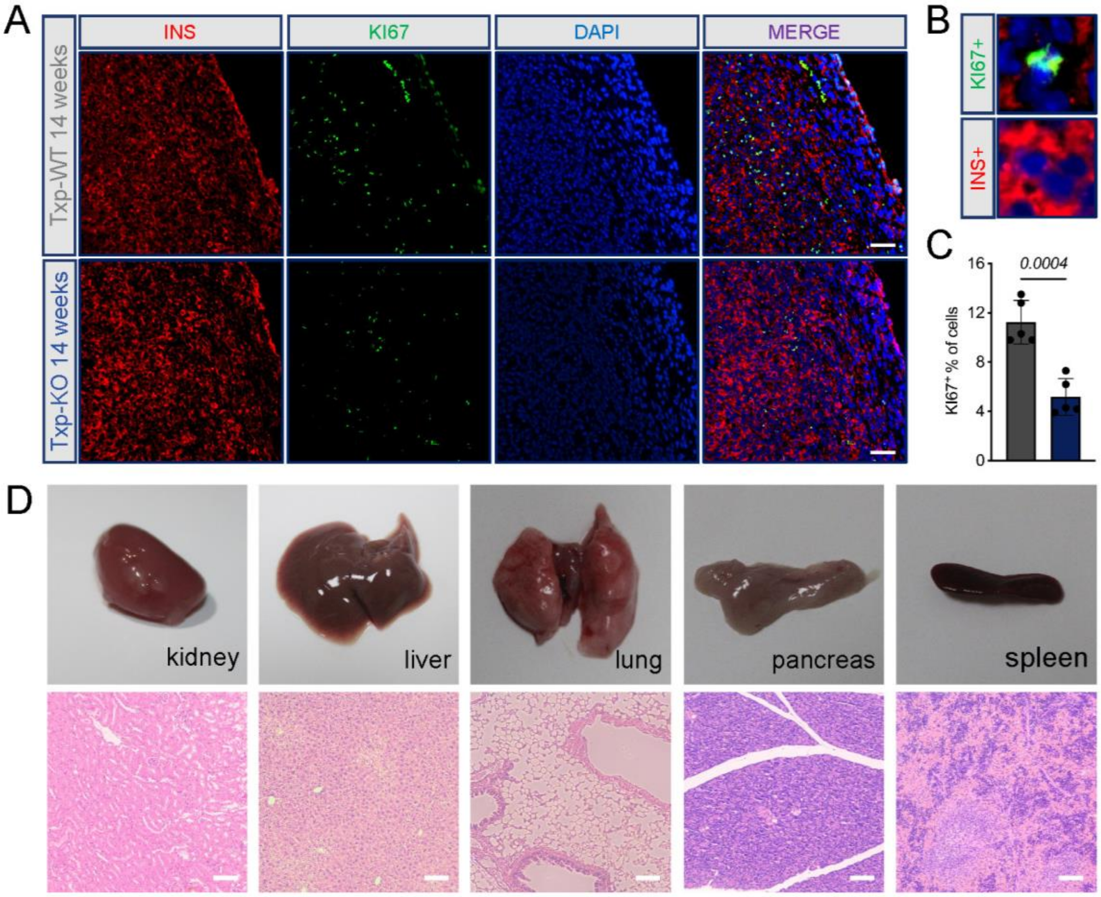

Figure S6. Additional analysis for grafts from diabetic mice transplanted with SC-β cells. (A) IF for INS/KI67 of grafts from mice transplanted with KO and WT β cells 14 weeks post-transplantation. Scale bars, 100 μm. (B) Representative IF for $KI67^+$ or INS^+ cells in (A). (C) Calculated percentage for KI67 positive PPs of grafts from mice transplanted with KO and WT β cells 14 weeks post-transplantation by morphological analysis of IF images of SC-β cells (n = 5) using ImageJ. Data presented as mean values ± SD. (D) Gross anatomy (top) and hematoxylin and eosin staining (bottom) of major organs (kidney, liver, lung, pancreas, and spleen). Scale bars, 100 μm. Staining results were repeated in at least three independent differentiations. All data are expressed as means ± SD. Statistical significance was calculated using two-tailed Student's t test.

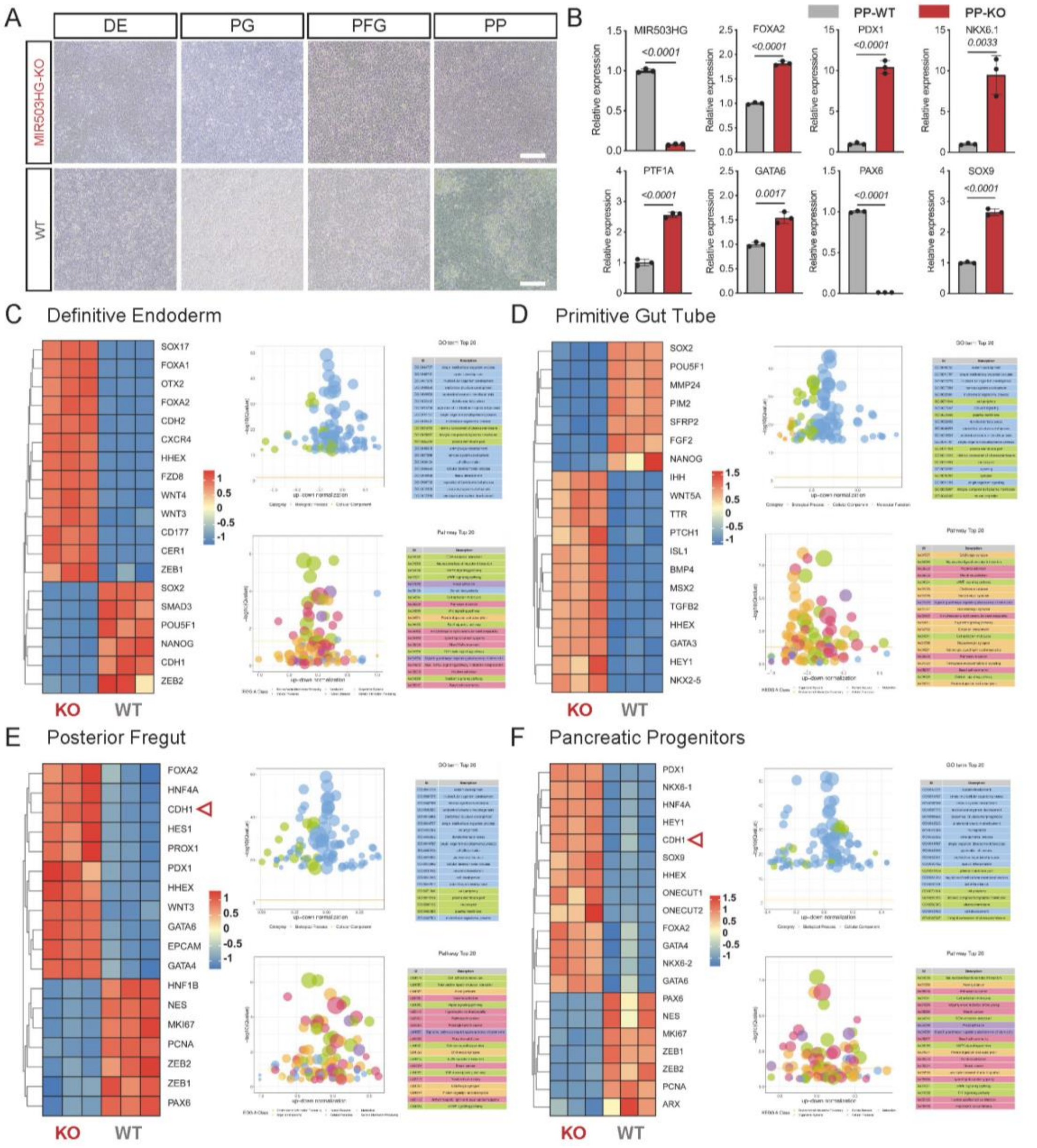

Figure S7. Additional analysis for WT and MIR503HG-KO PP cells. (A) Brightfield images of WT and MIR503HG-KO DE, PG, PFG, and PP. Scale bar, 500 μm. (B) qRT-PCR analysis of the expression level of MIR503HG, FOXA2, PDX1, NKX6.1, PTF1A, GATA6, PAX6, and SOX9 for WT and MIR503HG-KO PPs. (C–F) Heatmap, GO, and KEGG analysis for DEGs between WT and MIR503HG-KO cells at different stages (DE, PG, PFG, and PP) by mRNA-seq. Brightfield and qRT-PCR results were repeated in at least three independent differentiations. All data are expressed as means ± SD. Statistical significance was calculated using two-tailed Student's t-test.

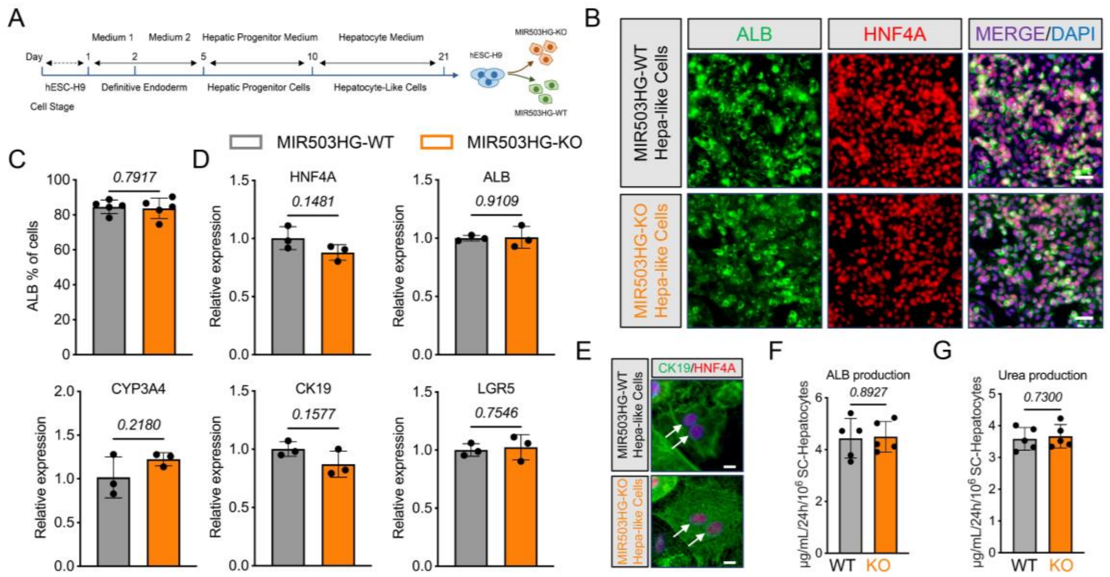

Figure S8. Analysis for WT and MIR503HG-KO hepatocytes. (A) Schematic representation of differentiation protocol imitating human hepatocytes development. (B) IF for ALB/HNF4A of WT and MIR503HG-KO SC-hepatocytes. Scale bar: 100 μm. (C) Calculated percentage for ALB positive hepatocytes by morphological analysis of IF images of SC-β cells (n = 6) using ImageJ. Data presented as mean values ± SD. (D) qRT-PCR analysis of different makers for hepatocytes. (E) IF for CK19/HNF4A of SC-hepatocytes. Scale bar: 100 μm. Arrowheads indicate hepatocytes with two nuclei. (F) ALB and (G) Urea production of WT and MIR503HG-KO SC-hepatocytes. Results were repeated in at least three independent differentiations. All data are expressed as means ± SD. Statistical significance was calculated using two-tailed Student's t-test.

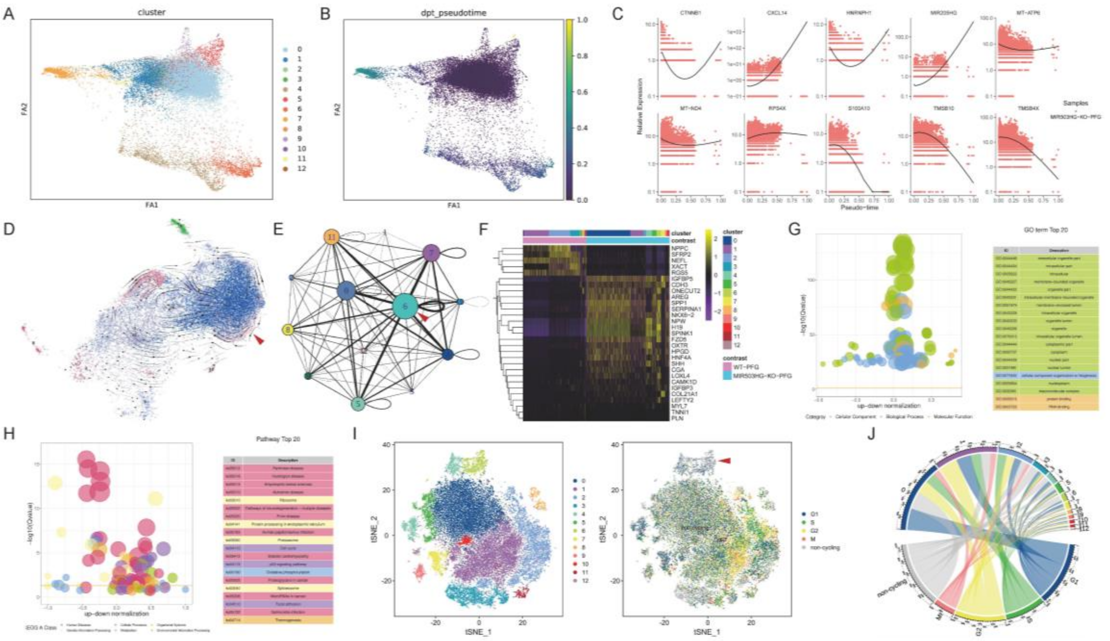

Figure S9. Additional analysis for WT and MIR503HG-KO PFG scRNA-seq data. (A) and (B) Pseudotime distribution of MIR503HG-KO PFGs. (C) Spline plots of representative gene expression dynamics for MIR503HG-KO PFGs. (D) *t*-SNE projection of the

MIR503HG-KO PFG clusters with RNA velocity vector overlays. Arrowhead denotes Cluster 6. (E) Significant ligand-receptor pairs between defined clusters. Circle sizes represent number of ligand-receptor pairs between each cell group and the edge width for communication probability. (F) Heatmap indicating top common DEGs of WT and MIR503HG-KO PFG cell clusters. (G and H) GO and KEGG analyses for top common DEGs of WT and MIR503HG-KO PFG cell clusters. (I) Cell cycles of WT and MIR503HG-KO PFG cell clusters. (J) Circle plot of cell cycles among defined WT and MIR503HG-KO PFG clusters.

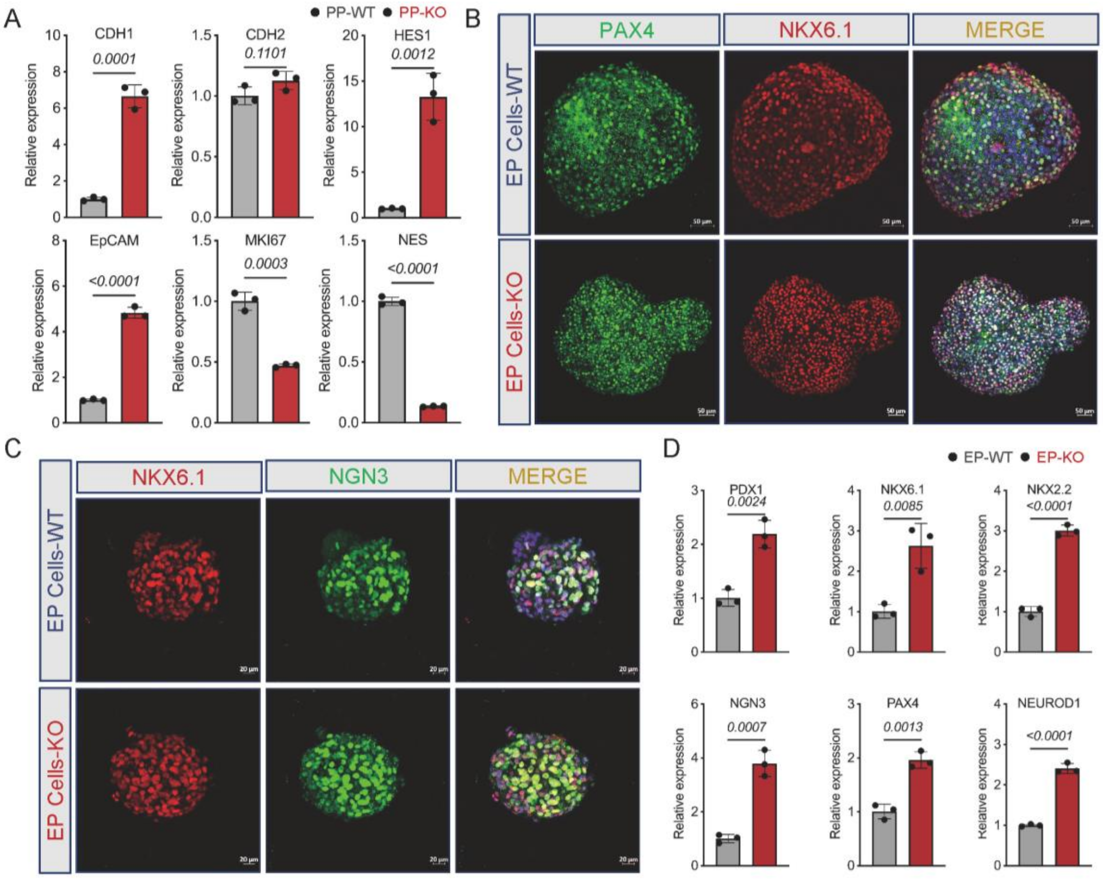

Figure S10. Additional analysis for WT and MIR503HG-KO PP cells and EPs. (A) qRT-PCR analysis of expression levels of CDH1, CDH2, HES1, EpCAM, MKI67, and NES for WT and MIR503HG-KO PPs. (B) IF for PAX4/NKX6.1 of WT and MIR503HG-KO EPs. Scale bars, 50 μm. (C) IF for PDX1/NGN3 of WT and MIR503HG-KO EPs. Scale bars, 20 μm. (D) qRT-PCR analysis of expression levels of PDX1, NKX6.1, NKX2.2, NGN3, PAX4, and NEUROD1 for WT and MIR503HG-KO EPs. Staining and qRT-PCR results were repeated in three independent differentiations. All data are expressed as means ± SD. Statistical significance was calculated using two-tailed Student’s t-test.

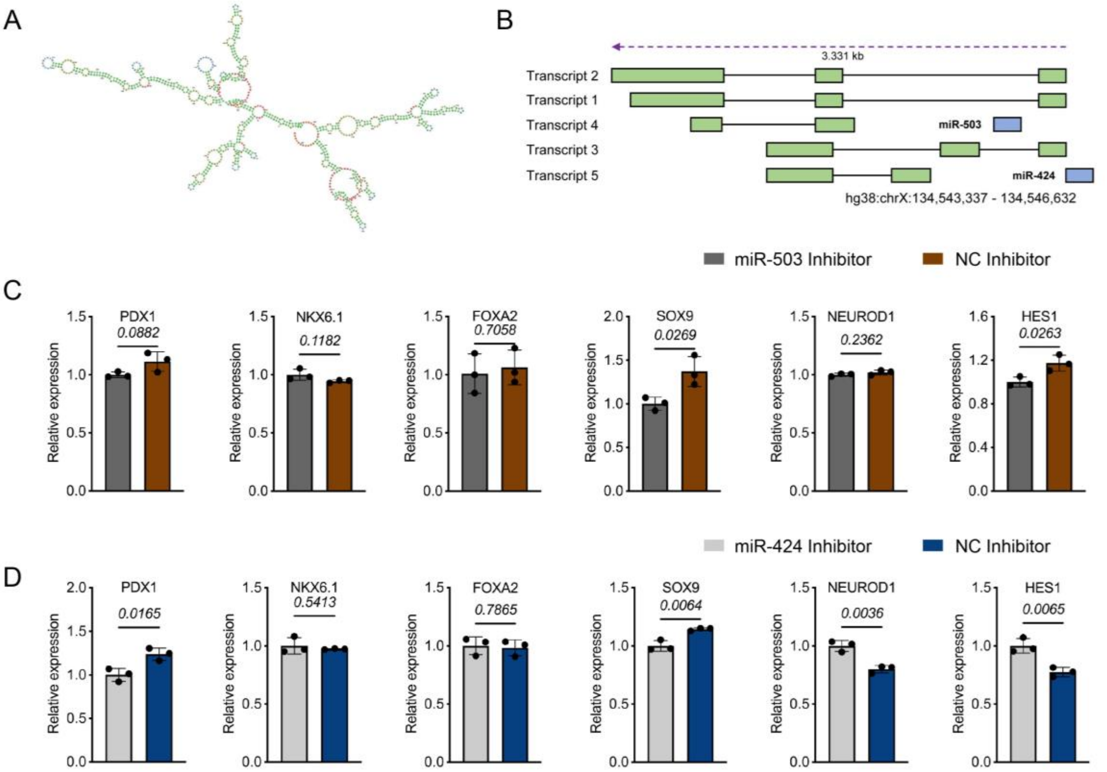

Figure S11. Additional MIR503HG functional analysis in pancreatic lineage specification. (A) Graphic illustration of the predicted MIR503HG secondary structure (LNCipedia, http://www.lncipedia.org). (B) Schematic representation of the MIR503HG, miRNA-424, and miRNA-503 loci according to GENCODE annotation (v26). (C and D) qRT-PCR analysis of expression levels of pancreatic development markers for PPs transfected with NC inhibitor, miRNA-503 inhibitor, or miRNA-424 inhibitor. qRT-PCR results were repeated in three independent differentiations. Statistical significance was calculated using two-tailed Student's t-test.

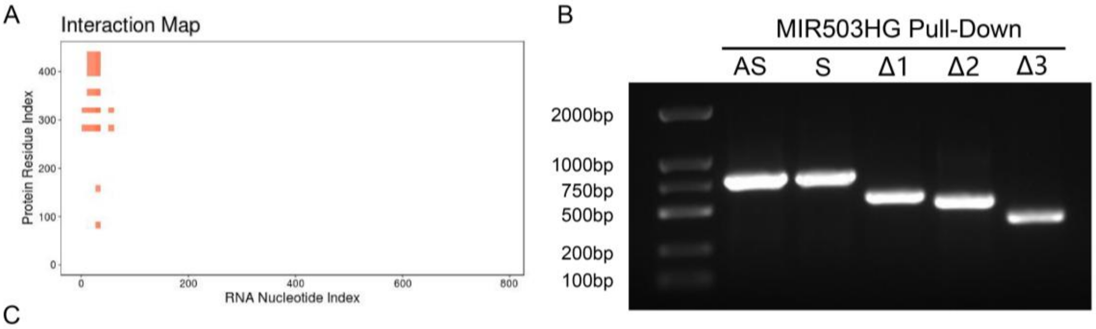

C

MIR503HG 1-200bp：

gaaggtagaaggtggggtctgccggacgcgtgttcctgccaccaggtgcccgctccccgcgaggccggctcag
gagcagaaggaagcccggtgccagccagccttcctgaaagaccaagcccgcgccatccggcttcctccagtgg
acgcctgcaggacccaggaatgtttttcttgaaggcatccagcatctccagtta

Figure S12. Additional analysis for the combined effect of MIR503HG and CtBP1. (A) Interaction map for binding regions of MIR503HG with CtBP1. (B) PCR identification of truncated MIR503HG. (C) Sequence of binding region of MIR503HG (1–200 bp) with CtBP1.

Table S1, Protocol for SC-β Cells differentiation

Stage	Days	Base medium	Factor	Final concentration
Stage 1 (Definitive Endoderm)	1	MCDB131	Activin A	100 ng/ml
			CHIR99021	6 μM
	2-4		Activin A	100 ng/ml
Stage 2 (Primitive Gut Tube)	5-7	MCDB131	KGF	50ng/ml
			SB431542	5 μM
			Vitamin C	0.25 mM
Stage 3 (Posterior Foregut)	8-10	MCDB131	KGF	50ng/mL
			LDN193189	200nM
			TPPB	500nM
			Retinoic acid	2μM
			SANT1	0.25μM
			Vitamin C	0.25 mM
Stage 4 (Pancreatic Progenitors)	11-15	DMEM	KGF	50ng/mL
			LDN193189	200 nM
			TPPB	500 nM
			Retinoic acid	0.1μM
			SANT1	0.25 μM
			Vitamin C	0.25 mM
Stage 5 (Endocrine Progenitors)	16-20	DMEM	Alk5i-II	10 uM
			LDN193189	0.3 uM
			T3	1 uM
			SANT1	0.25μM
			Heparin	10 ug/mL
			γ-secretase inhibitor Xxi	0.1 uM
			Retinoic acid	0.1 μM
			Vitamin C	0.25 mM
Stage 6 (SC-β Cells)	21-35	DMEM	Trolox	10 μM
			Heparin	10 μg/ml
			T3	1 μM
			Alk5i-II	10 μM
			R428	0.5 μM
			Forskolin	10 μM
			Zinc sulfate	10 μM
			N-Acetyl-L-cysteine	2 mM

Table S2. sgRNA and sequencing primers of mutant cell lines

	sgRNA (5'-3')		Forward Primer Sequence		Reverse Primer Sequence
MIR 503HG$^{-/-}$-3	AATGAAGGGGGATCGATCTGGGG	F1	GGACAATGAATGAGACTTGTAATATGC	R1	CAGGGAGATACACGTTTGCTACAC
MIR 503HG$^{-/-}$-4	ATTTGCCTTGATGAGTTGATAGG	F2	GAACCTCAACTGATTTGAACAGATG	R3	CTTTCCTGAATTCTTAAGTCCCTTC

Table S3. Primer sequences used for gene expression by qRT-PCR.

Primer name	Orientation	Sequence(5'-3')
NKX6.1	Forward	GGACTGCCACGCTTTAGCA
	Reverse	TGGGTCTCGTGTGTTTTCTCT
PDX1	Forward	ATCTCCCCATACGAAGTGCC
	Reverse	CGTGAGCTTTGGTGGATTTCAT
NEUROG3	Forward	CTAAGAGCGAGTTGGCACTGA
	Reverse	GAGGTTGTGCATTCGATTGCG
FOXA2	Forward	GGAGCAGCTACTATGCAGAGC
	Reverse	CGTGTTCATGCCGTTCATCC
NEUROD1	Forward	ATGACCAAATCGTACAGCGAG
	Reverse	GTTCATGGCTTCGAGGTCGT
SOX9	Forward	AGCGAACGCACATCAAGAC
	Reverse	CTGTAGGCGATCTGTTGGGG
GATA4	Forward	GTGTCCCAGACGTTCTCAGTC
	Reverse	GGGAGACGCATAGCCTTGT
GATA6	Forward	CTCAGTTCCTACGCTTCGCAT
	Reverse	GTCGAGGTCAGTGAACAGCA
ISL1	Forward	GCGGAGTGTAATCAGTATTTGGA
	Reverse	GCATTTGATCCCGTACAACCT
MAFA	Forward	GAGCGGCTACCAGCATCAC
	Reverse	CTCTGGAGTTGGCACTTCTCG
PAX4	Forward	AGTCCTGCGGGCATTACAG
	Reverse	GGGAGAAGATAGTCCGATTCCG
PAX6	Forward	TGGGCAGGTATTACGAGACTG
	Reverse	ACTCCCGCTTATACTGGGCTA
UCN3	Forward	GGAGGGAAGTCCACTCTCG
	Reverse	TGTAGAACTTGTGGGGGAGG
NKX2.2	Forward	CCGGGCCGAGAAAGGTATG
	Reverse	GTTTGCCGTCCCTGACCAA
IAPP	Forward	TCTCATCGCAGTCAGGATCATAA
	Reverse	TCATTGTGCTCTCTGTTGCAT
PTF1A	Forward	TTCACCGACCAGTCTTCACG
	Reverse	GTGGCTAAGGAACTCCACCT
Insulin	Forward	GCAGCCTTTGTGAACCAACAC
	Reverse	CCCCGCACACTAGGTAGAGA
GCG	Forward	CTGAAGGGACCTTTACCAGTGA
	Reverse	CCTGGCGGCAAGATTATCAAG
SST	Forward	ACCCAACCAGACGGAGAATGA
	Reverse	GCCGGGTTTGAGTTAGCAGA
MIR503HG	Forward	CTTGAAGGCATCCAGCATCTC
	Reverse	TTGGGACACTTGGGTGGTTTT
CTBP1	Forward	CGACCCTTACTTGTCGGATGG
	Reverse	TTGACGGTGAAGTCGTTGATG
CDH1	Forward	AAGTGCTGCAGCCAAAGACAGA
	Reverse	AGGTAGACCCACCTCAATCATCCTC
CDH2	Forward	TGGACCATCACTCGGCTTA
	Reverse	ACACTGGCAAACCTTCACG
HES1	Forward	GGCTGGAGAGGCGGCTAA
	Reverse	GAGAGGTGGGTTGGGGAGTT
EPCAM	Forward	AATCGTCAATGCCAGTGTACTT
	Reverse	TCTCATCGCAGTCAGGATCATAA
MKI67	Forward	ACGCCTGGTTACTATCAAAAGG
	Reverse	CAGACCCATTTACTTGTGTTGGA
NES	Forward	CTGCTACCCTTGAGACACCTG
	Reverse	GGGCTCTGATCTCTGCATCTAC
CHIP-CDH1	Forward	GTAAAAGCCCTTTCTGATCCCAGG
	Reverse	TCACAGGTGCTTTGCAGTTCCGA

CHIP-HES1	Forward	GCGCACACACACTCACTCA
	Reverse	ACCACCCTTTCCCTTTTCAT
GAPDH	Forward	ACGAACGGATTAACAGGGTCA
	Reverse	GGCTGTTGTCATACTTCTCATGG
OCT4	Forward	GGTTCTCGATACTGGTTCGC
	Reverse	GTGGAGGAAGCTGACAACAA
NANOG	Forward	ATGGAGGAGGGAAGAGGAGA
	Reverse	GATTTGTGGGCCTGAAGAAA
SOX2	Forward	TACAGCATGTCCTACTCGCAG
	Reverse	GAGGAAGAGGTAACCACAGGG
ALB	Forward	TTTATGCCCCGGAACTCCTT
	Reverse	AGTCTCTGTTTGGCAGACGAA
HNF4A	Forward	GGCCAAGTACATCCCAGCTTT
	Reverse	CAGCACCAGCTCGTCAAGG
LGR5	Forward	GACTTTAACTGGAGCACAGA
	Reverse	AGCTTTATTAGGGATGGCAA
CYP3A4	Forward	CTTCATCCAATGGACTGCATAAAT
	Reverse	TCCCAAGTATAACACTCTACACAGACAA
CK19	Forward	CGCGGCGTATCCGTGTCCTC
	Reverse	AGCCTGTTCCGTCTCAAACTTGGT

Table S4. Antibodies list.

Antibody Target	Company	Catalog number	Dilution ratio
Mouse anti-FOXA2	Novus	H00003170-M12	IF, 1:200
Rabbit anti-HNF1A	CST	89670S	IF, 1:100
Rabbit anti-PDX1	Abcam	ab219207	IF, 1:100, WB, 1:1000
Rabbit anti-NKX6.1	CST	5451S	IF, 1:100
Rabbit anti-NANOG	Abcam	ab109250	IF, 1:100
Rabbit anti-OCT4	Proteintech	11263-1-AP	IF, 1:100
Rabbit anti-SOX2	Proteintech	11064-1-AP	IF, 1:100
Mouse anti-SSEA4	Abcam	ab16287	IF, 1:100
Mouse anti-KI67	Abcam	ab279653	IF, 1:100
Rabbit anti-NKX6.1	Abcam	ab221549	WB, 1:1000
Rabbit anti-Insulin	CST	3014S	IF, 1:100
Mouse anti-PDX1	BD	562160	IF, 1:100
Rabbit anti-C-Peptide	CST	4593S	IF, 1:100
Rabbit anti-SOX9	Abcam	ab185966	IF, 1:100
Rabbit anti-E-CAD	Abcam	Ab40772	IF, 1:100, WB, 1:1000

Mouse anti-EPCAM	Abcam	ab23582	IF, 1:100, WB, 1:1000
Rabbit anti-CtBP1	CST	8684S	IF, 1:100, WB, 1:1000, CHIP, 1:100
Rabbit anti-HES1	Abcam	Ab108937	IF, 1:200, WB, 1:1000
Mouse anti-YAP1	Proteintech	66900-1-Ig	IF, 1:100, WB, 1:1000
Mouse anti-Glucagon	R&D Systems	MAB1249	IF, 1:200
Mouse anti-OCT-3/4	BD	561555	IF, 1:100
Mouse anti-E-CAD	Abcam	ab231303	IF, 1:100
Rabbit anti-FOXA2	Proteintech	22474-1-AP	IF, 1:100
Rabbit anti-MAFA	CST	79737S	IF,1:200
Mouse anti-NKX6.1	BD	563022	IF, 1:100
Goat anti-mouse Alexa Flour 555	Abcam	Ab150114	IF, 1:200
Goat anti-rabbit Alexa Flour 488	Abcam	Ab150077	IF, 1:200
Rabbit anti-phospho-YAP (Ser127)	CST	13008S	WB, 1:1000
Mouse anti-Vimentin	Proteintech	60330-1-IG	WB, 1:1000
Mouse anti-SNAI2	Proteintech	12129-1-AP	WB, 1:1000
Goat anti-mouse immunoglobulin G (IgG) (H+L)	Jackson ImmunoResearch	115-035-003	WB, 1:20000
Goat anti-rabbit IgG (H+L)	Jackson ImmunoResearch	111-035-003	WB, 1:20000
Rabbit anti-SOX9	CST	82630S	WB, 1:1000
Rabbit anti-GAPDH	Abcam	Ab181602	WB, 1:1000
Mouse APC-conjugated anti-PDX-1	R&D Systems	IC2419A	FCM, 5ul/test
Alexa Flour® 647 Mouse Anti-Insulin	BD	565689	FCM, 5ul/test
Alexa Fluor® PE Mouse anti-NKX6.1	BD	563023	FCM, 5ul/test
Rabbit anti-Albumin	Abcam	Ab207327	IF, 1:200
Rabbit anti-CK19	Abcam	Ab52625	IF, 1:200
Mouse anti-HNF4A	R&D Systems	MAB4605	IF, 1:100
PerCP-Cy™ 5.5 Mouse anti-PDX1	BD	563436	FCM, 5ul/test
Pharmingen™ PE Mouse Anti-Glucagon	BD	565860	FCM, 5ul/test
